# Immunohistochemical study of the phenotypic change of the mesenchymal cells during portal tract maturation in normal and fibrous (ductal plate malformation) fetal liver

**DOI:** 10.1186/1476-5926-8-5

**Published:** 2009-07-14

**Authors:** Julien Villeneuve, Fanny Pelluard-Nehme, Chantal Combe, Dominique Carles, Christine Chaponnier, Jean Ripoche, Charles Balabaud, Paulette Bioulac-Sage, Sébastien Lepreux

**Affiliations:** 1 INSERM U889, Université Bordeaux2, F-33076 Bordeaux, France; 2 Service d'Anatomie Pathologique, Hôpital Pellegrin, F-33076 Bordeaux, France; 3 Département de Pathologie et d'Immunologie, CMU, Genève, Suisse

## Abstract

**Background:**

In adult liver, the mesenchymal cells, portal fibroblasts and vascular smooth muscle cells can transdifferentiate into myofibroblasts, and are involved in portal fibrosis. Differential expression of markers, such as alpha-smooth muscle actin (ASMA), h-caldesmon and cellular retinol-binding protein-1 allows their phenotypic discrimination. The aim of our study was to explore the phenotypic evolution of the mesenchymal cells during fetal development in normal liver and in liver with portal fibrosis secondary to ductal plate malformation in a series of Meckel-Gruber syndrome, autosomal recessive polycystic kidney disease and Ivemark's syndrome.

**Results:**

At the early steps of the portal tract maturation, portal mesenchymal cells expressed only ASMA. During the maturation process, these cells were found condensed around the biliary and vascular structures. At the end of maturation process, only cells around vessels expressed ASMA and cells of the artery tunica media also expressed h-caldesmon. In contrast, ASMA positive cells persisted around the abnormal biliary ducts in fibrous livers.

**Conclusion:**

As in adult liver, there is a phenotypic heterogeneity of the mesenchymal cells during fetal liver development. During portal tract maturation, myofibroblastic cells disappear in normal development but persist in fibrosis following ductal plate malformation.

## Introduction

In the liver, different fibrocompetent cells have been described in accordance with their topography, their morphology and their main functions: portal fibroblasts and vascular smooth muscle cells in the portal tract; hepatic stellate cells (HSC) and "second layer cells" around the centrolobular veins in lobular area (review in Guyot et al [1]). The heterogeneity of these fibrocompetent cells is characterised by the expression of different markers. For example, quiescent HSC express cellular retinol-binding protein-1 (CRBP-1) but not alpha-smooth muscle actin (ASMA) or h-caldesmon [2-5]. Vascular smooth muscle cells expressed ASMA and h-caldesmon [6]. Finally, portal fibroblasts expressed neither ASMA nor CRBP-1, but expressed vimentin [3,4]. Myofibroblasts are absent in the normal liver but, during liver fibrosis, these cells can acquire a myofibroblastic phenotype, notably by the expression of ASMA [1,7].

The phenotypic evolution of mesenchymal cells during the fetal human liver development has not been studied with the markers discussed above. The mesenchymal cells derived from the stroma of the septum transversum which is invaded by epithelial cell clusters from hepatic diverticulum during the 4^th ^week of development (WD) [8]. The lobulation of the fetal liver begin near the liver hilum at the 9^th ^WD, and progresses from the hilum to the periphery of the liver until at about 1-month post partum. Concerning the future lobular area, HSC and the second layer cells around the centrolobular veins, derive from mesenchymal cells, as well as the mesenchymal vessels which formed the primitive hepatic sinusoids [9,10]. Concerning the portal tract, its centrifugal development is closely associated with intra-hepatic biliary tree development [11]. Depending exclusively on the location of the portal tract along the portal tract tree, between the hilum and the periphery, the sequence of maturation of a portal tract schematically comprises 3 stages [12]: 1) At the ductal plate stage, segments of double-layered cylindrical or tubular structures, called ductal plate, outlined the future portal tract. The future portal tract contains also large portal vein branch and limited stroma; 2) At the ductal plate remodelling stage, the tubular structures become incorporated into the stroma surrounding the portal vein branch and the rest of the ductal plate involutes. Arterial branches are also present; 3) At the remodelled stage, the portal tract is mature: it contains a branch of the portal vein, two branches of the hepatic artery and two bile ducts [13]. In cases of ductal plate malformation, notably observed in Ivemark's renal-hepatic-pancreatic dysplasia or Ivemark's dysplasia syndrome type II (IDS2), in Meckel-Gruber syndrome (MKS) and in autosomal recessive polycystic kidney disease (ARPKD), the portal tract was deeply modified [14-16]. It was characterised by portal tract fibrosis, more mesenchymal cells with ASMA expression and increased number of arteries [11,17].

The aims of our study were to follow principally the ASMA, h-caldesmon, CRBP-1 expression of mesenchymal cells during the normal development of the fetal liver and to explore the phenotypic evolution of the portal tract mesenchymal cells during the abnormal development of fetal liver presenting fibrosis following ductal plate malformation.

## Results

### Normal fetal liver – Histology

In all tissue samples, the fetal liver tissues showed anastomosing sheets of fetal hepatocytes. Each sheet, being two or several cells in thickness, was separated from the others by capillaries. Haematopoiesis was present in all cases and prominent in the capillary lumen or in the Disse space after 12 WD. After 11 WD, future portal tracts appeared in the parenchyma and developed with a centrifugal manner from the hilum to the periphery of the liver. Depending on the tissue section level (near the hilum or at the periphery), the 3 portal tract maturation stages (described above) were present. In the parenchyma, future centrolobular veins with a thin wall were present.

### Normal fetal liver – Immunohistochemistry

#### Alpha-smooth muscle actin (ASMA)

**Figure 1:**
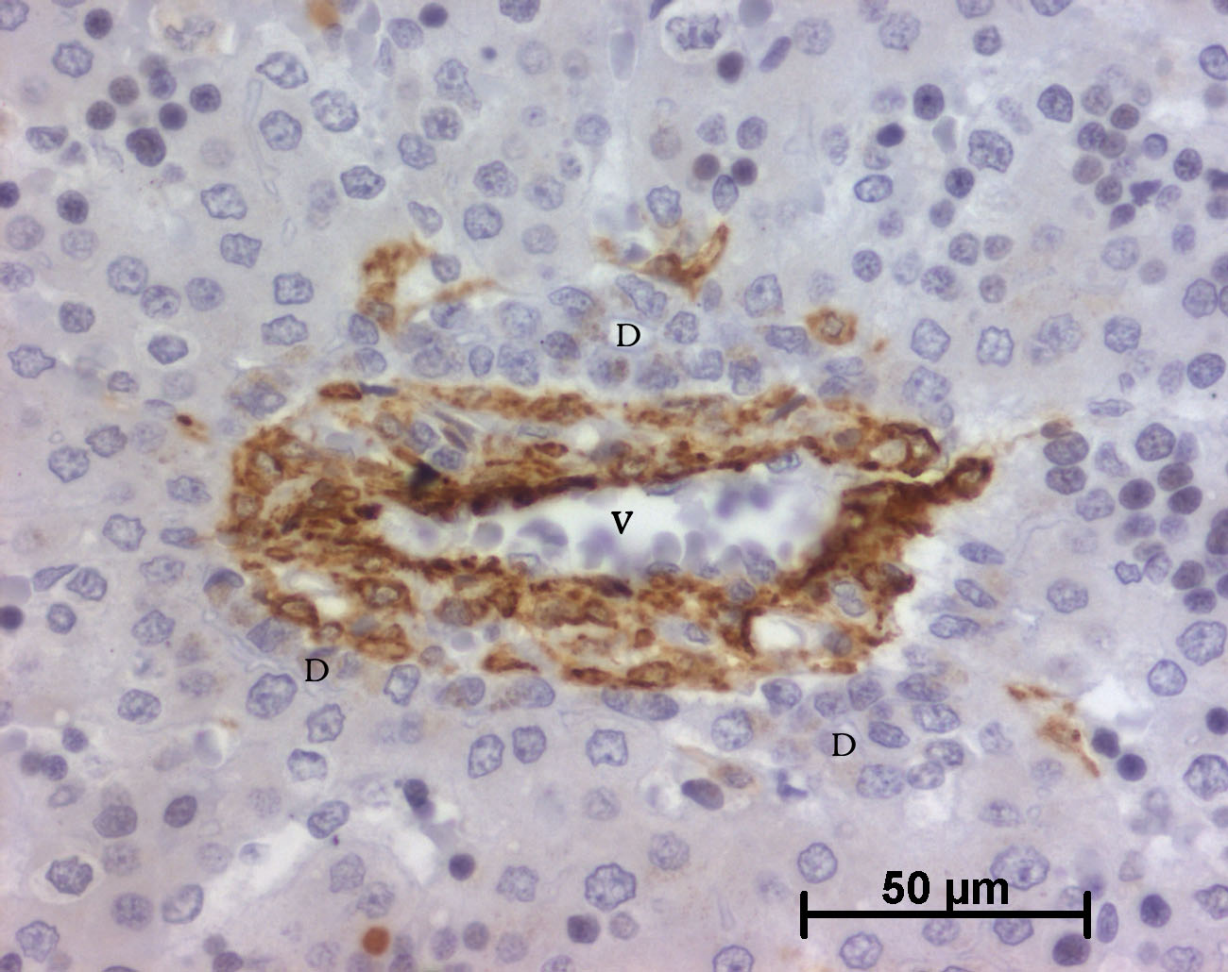
**Alpha-smooth muscle actin (ASMA) expression in normal fetal liver**. At the ductal plate stage, all fusiform cells in the portal stroma express ASMA (15 WD) (V: portal vein; D: ductal plate).

**Figure 2:**
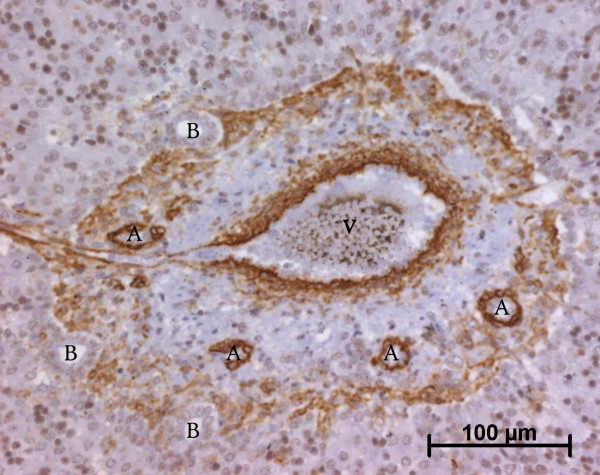
**Alpha-smooth muscle actin (ASMA) expression in normal fetal liver**. At the remodelling stage, fusiform cells at distance of the vessels and the biliary structures are ASMA negative (13 WD) (V: portal vein; A: artery; B: bile duct).

**Figure 3:**
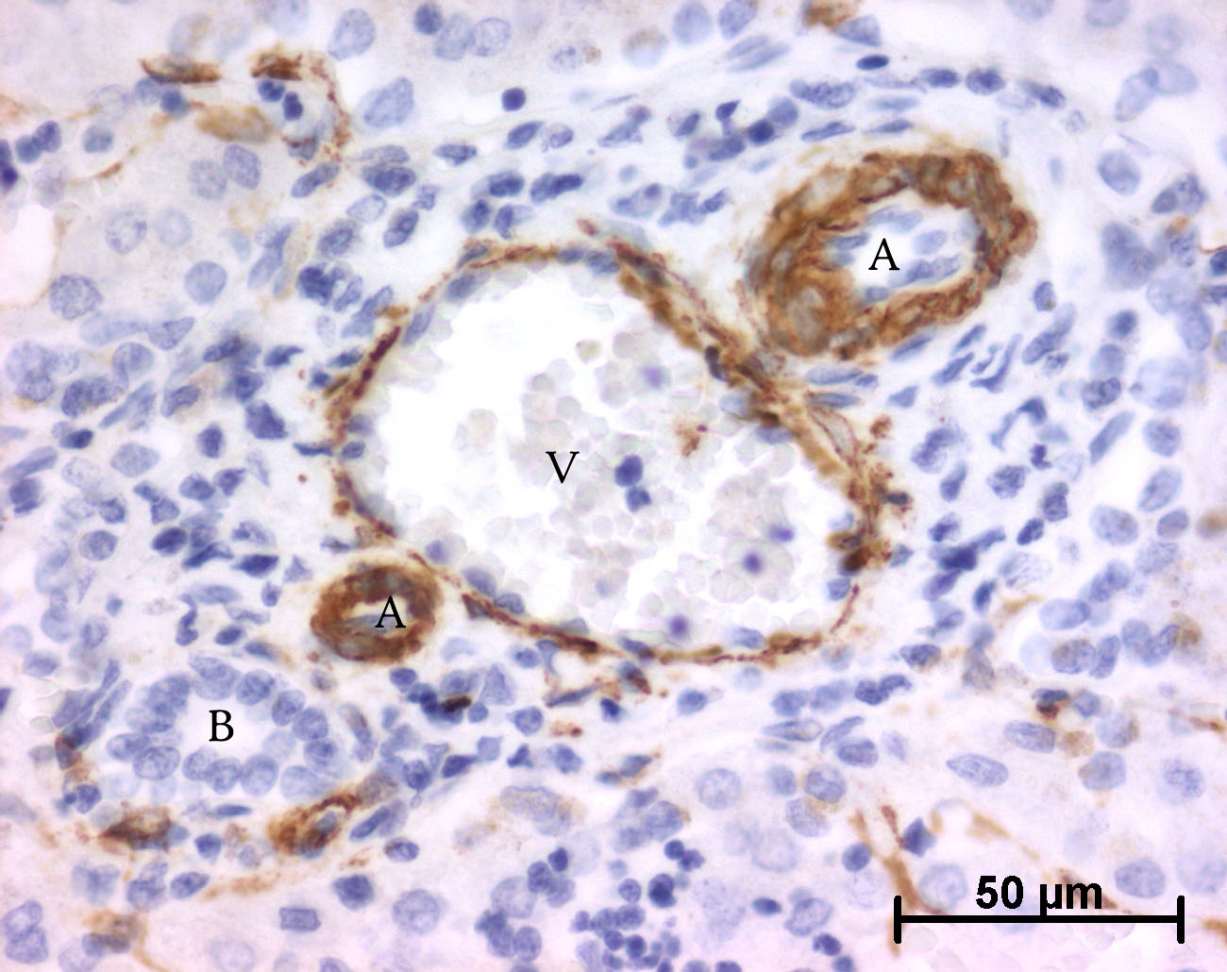
**Alpha-smooth muscle actin (ASMA) expression in normal fetal liver**. At the remodelled stage, ASMA expression in portal tract is confined to the tunica media of vessels (20 WD) (V: portal vein; A: artery; B: bile duct).

**Figure 4:**
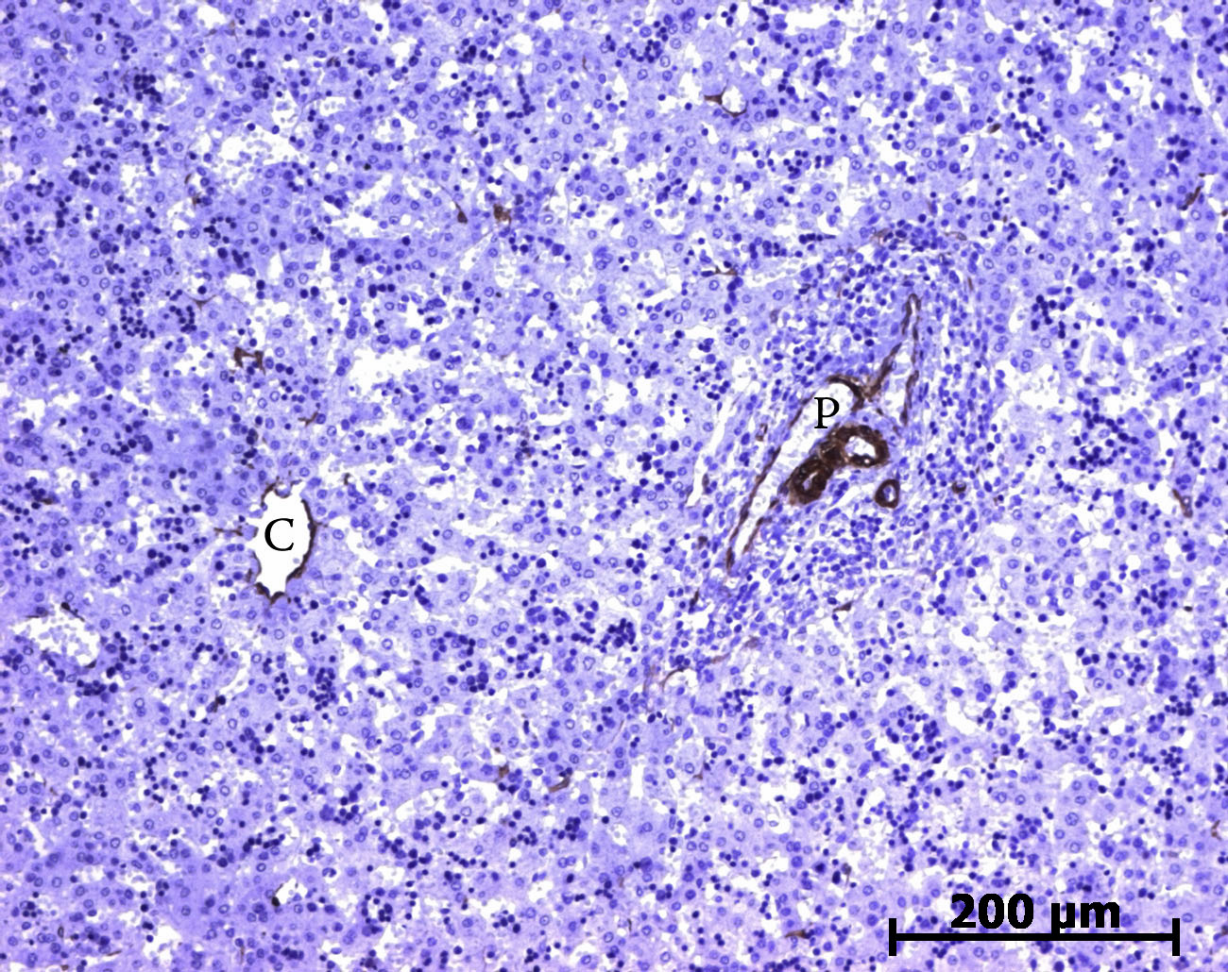
**Alpha-smooth muscle actin (ASMA) expression in normal fetal liver**. Rare cells are stained with ASMA within the lobule (23 WD) (C: centrolobular vein; P: portal tract).

**Figure 5:**
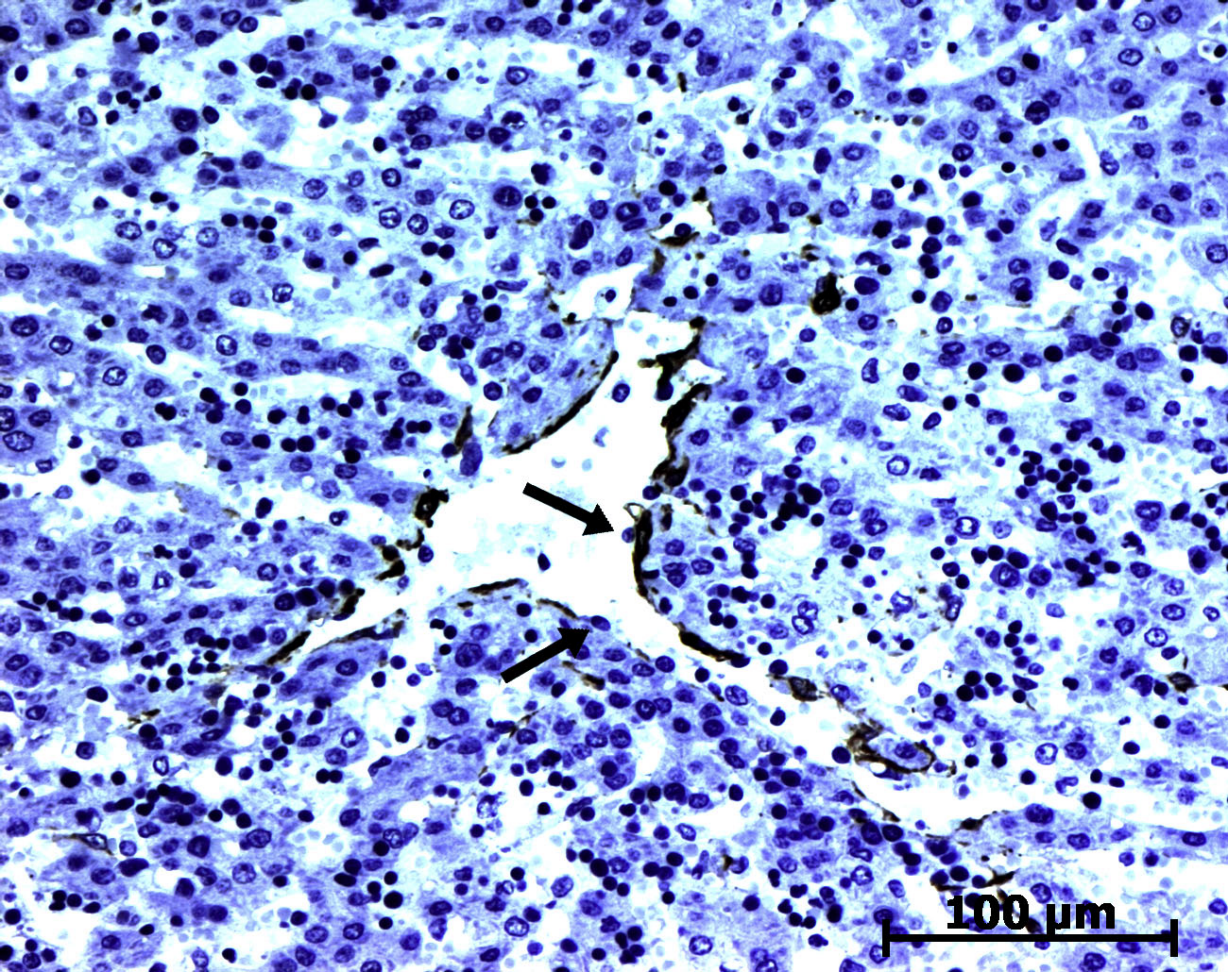
**Alpha-smooth muscle actin (ASMA) expression in normal fetal liver**. Second layer cells around the centrolobular vein express ASMA, but not endothelial cells (arrows) (23 WD).

At the ductal plate stage, all fusiform cells in the stroma between endothelial cells of the future portal vein and the first plate of hepatoblasts expressed ASMA (Figure 1). At the remodelling stage (Figure 2), in addition with fusiform cells under the endothelium of the portal vein and cells in the tunica media of arteries, fusiform cells around the tubular biliary structures enmeshed in the portal stroma and the fusiform cells close to the ductal plate remnants expressed ASMA. The fusiform cells at distance of these two areas were negative for ASMA expression. At the remodelled stage, ASMA expression was restricted to the cells in the tunica media of the portal vessels (Figure 3). After 20 WD, a few fusiform cells scattered around large bile ducts in the large portal tracts near the hilum also expressed ASMA. Concerning the lobular area, rare stained HSC were scattered in the parenchyma (Figure 4); only 3 cases (3/28 cases), respectively at the 13^th^, 16^th ^and 21^th ^WD, showed foci of stained HSC. Cells around terminal venules near the portal tract and fusiform cells around centrolobular veins expressed ASMA (Figure 5). Hepatocytic cells were not stained.

**Figure 6:**
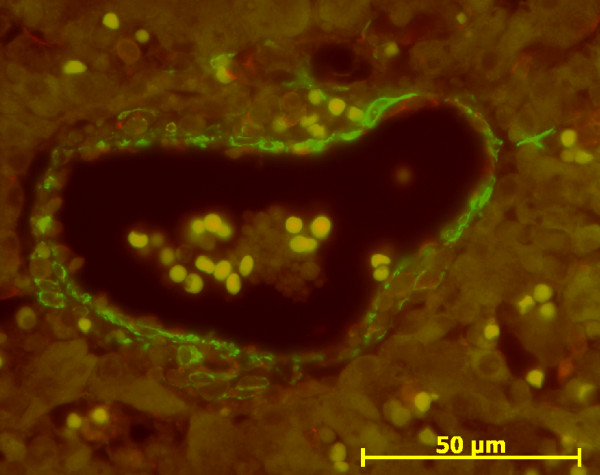
**Double immunofluorescence with ASMA (green)/vimentin (red) in normal fetal liver**. At the ductal plate stage, mesenchymal cells around portal vein express ASMA (green) (13 WD).

**Figure 7:**
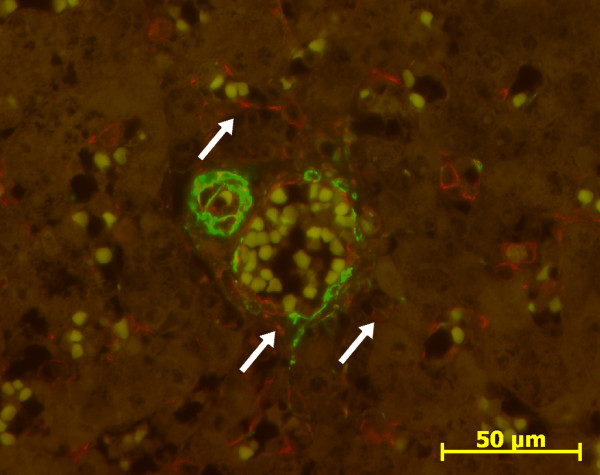
**Double immunofluorescence with ASMA (green)/vimentin (red) in normal fetal liver**. At the remodelled stage, cells around portal vein and artery express ASMA (green), and portal fibroblasts (arrows) express only vimentin (red) (31 WD).

With double immunofluorescence using anti ASMA and anti vimentin antibodies, negative ASMA fusiform cells within the portal tract notably at the remodelled stage expressed only vimentin (Figures 6 and 7). Endothelial cells of the portal tract vessels, HSC and Kupffer cells were also stained, as previously described in adult liver [4,18].

#### h-Caldesmon

**Figure 8:**
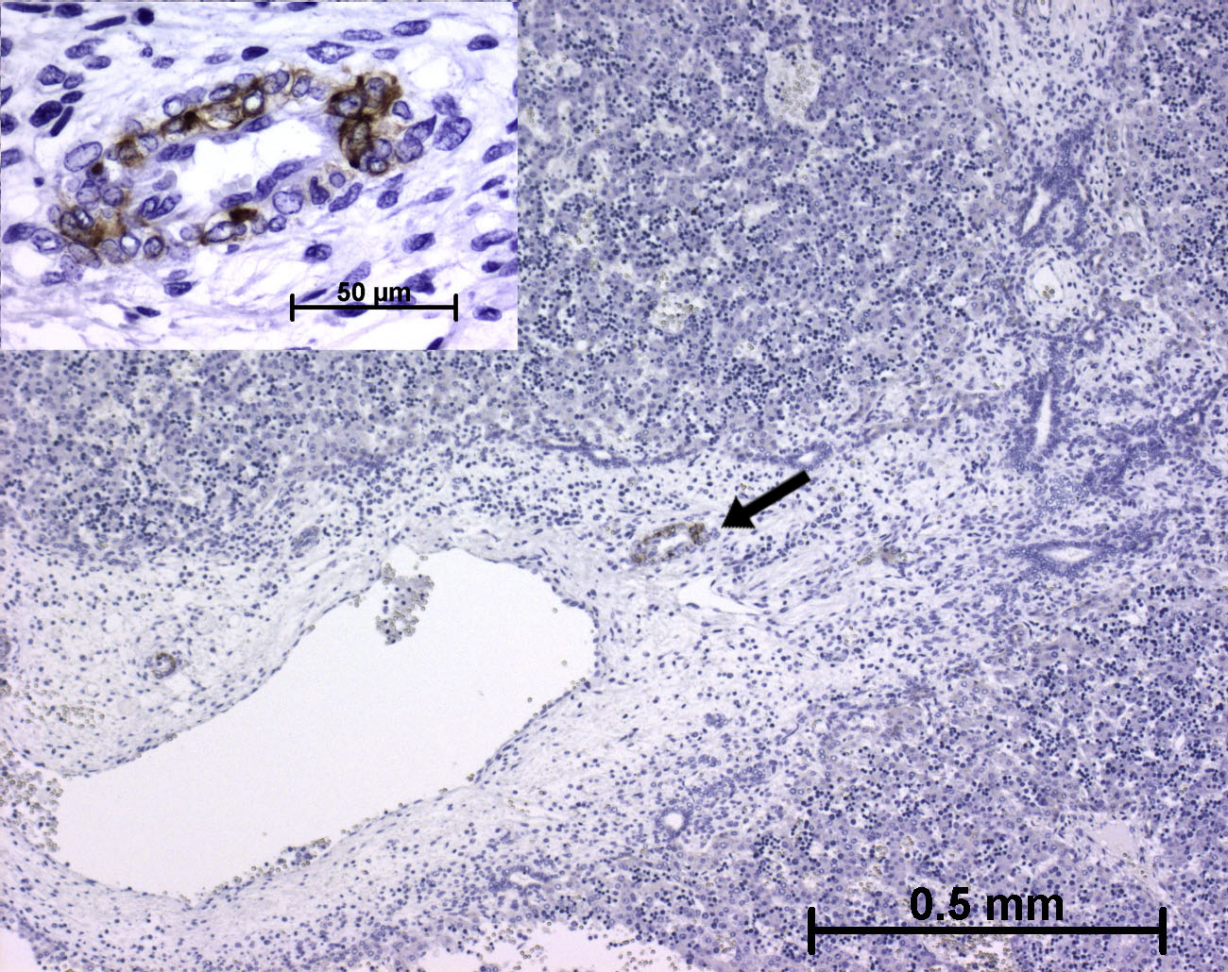
**h-Caldesmon expression in normal fetal liver**. At the early time of development, the arterial tunica media cells in the hilum express h-caldesmon (arrow and left insert) (11 WD).

**Figure 9:**
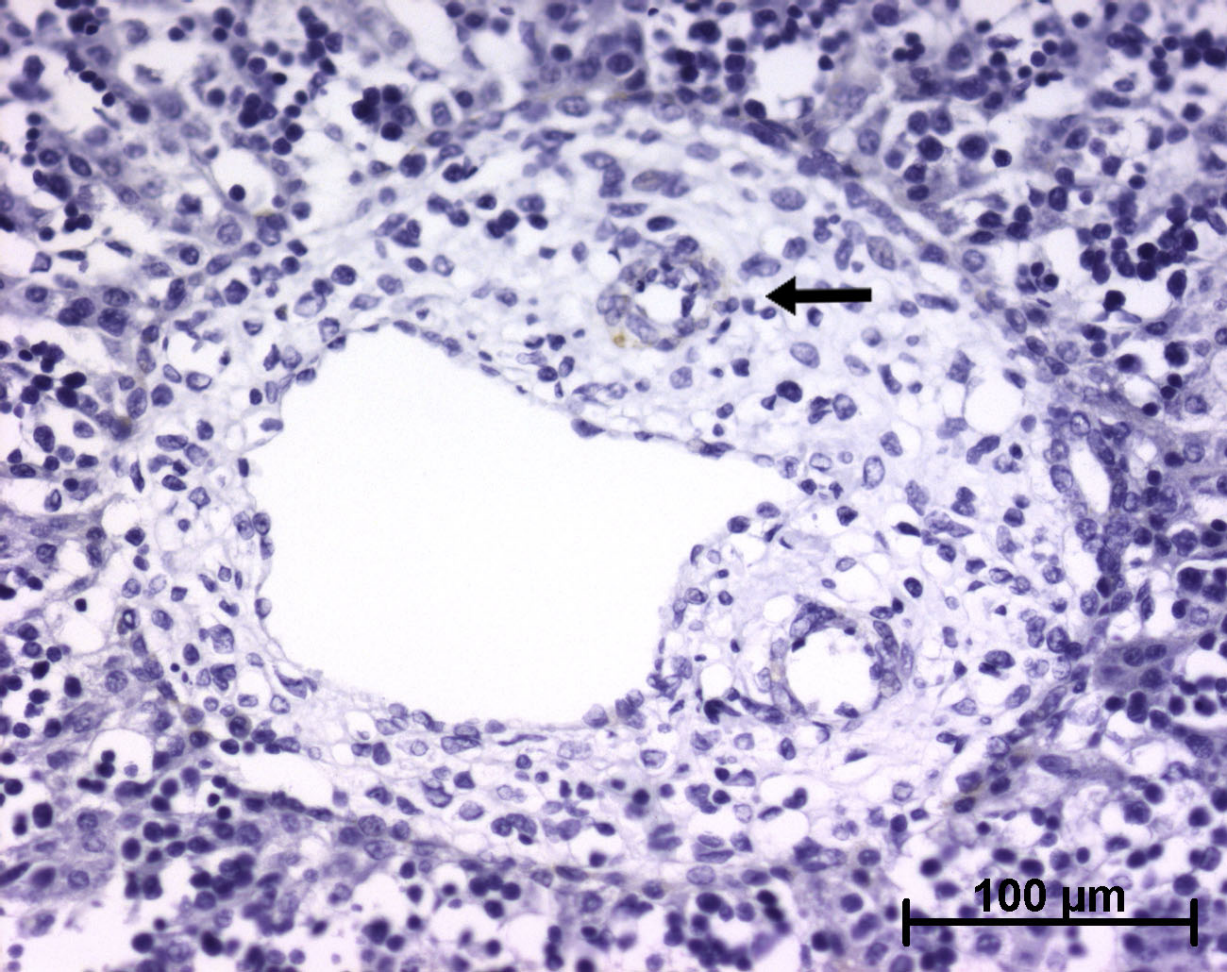
**h-Caldesmon expression in normal fetal liver**. During the early time of the ductal plate remodelling, h-caldesmon is not detected in cells around the portal arterial branch (arrow) (11 WD).

**Figure 10:**
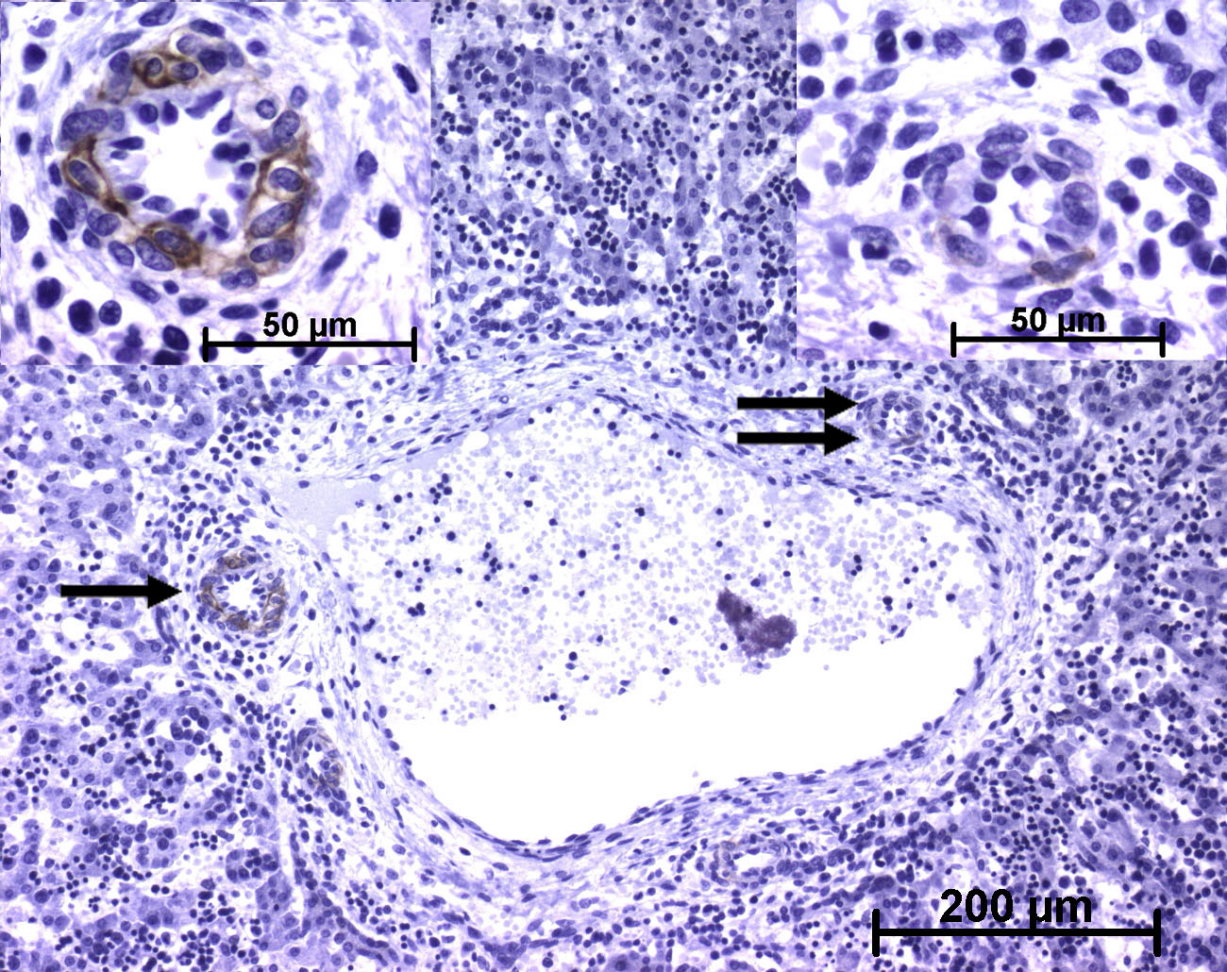
**h-Caldesmon expression in normal fetal liver**. At advanced time in the remodelling stage, the arterial tunica media cells express faintly h-caldesmon (double arrow, right insert) or more strongly (single arrow, left insert) (13 WD). Whatever the stage of portal tract maturation, interstitial stromal cells are not stained.

**Figure 11:**
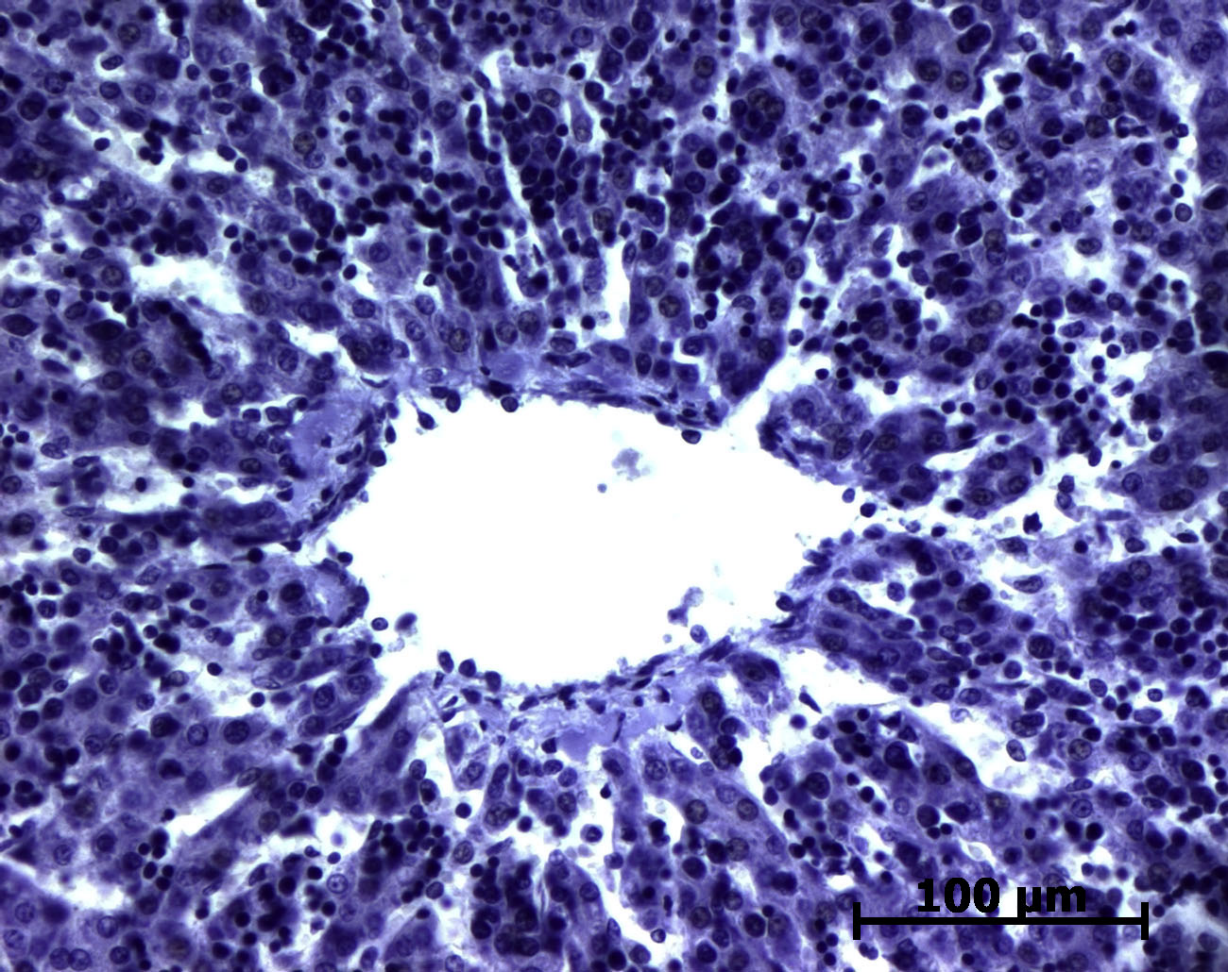
**h-Caldesmon expression in normal fetal liver**. Around the centrolobular cells, no h-caldesmon expression is found (23 WD).

h-Caldesmon, a specific marker for the smooth muscle cell differentiation last step [6,19], was expressed at 11 WD in the arterial tunica media of the hilum (Figure 8). At the ductal plate stage, after the 11 WD, h-caldesmon was not expressed in the future portal tracts. At the remodelling stage, h-caldesmon expression was variably present in fusiform cells of the arterial tunica media (Figures 9 and 10). At the remodelled stage, all the cells in the arterial tunica media were stained. Whatever the stage, the other portal cells, as well as cells in the lobular area, did not express h-caldesmon (Figure 11).

#### Cellular retinol-binding protein-1 (CRBP-1)

**Figure 12:**
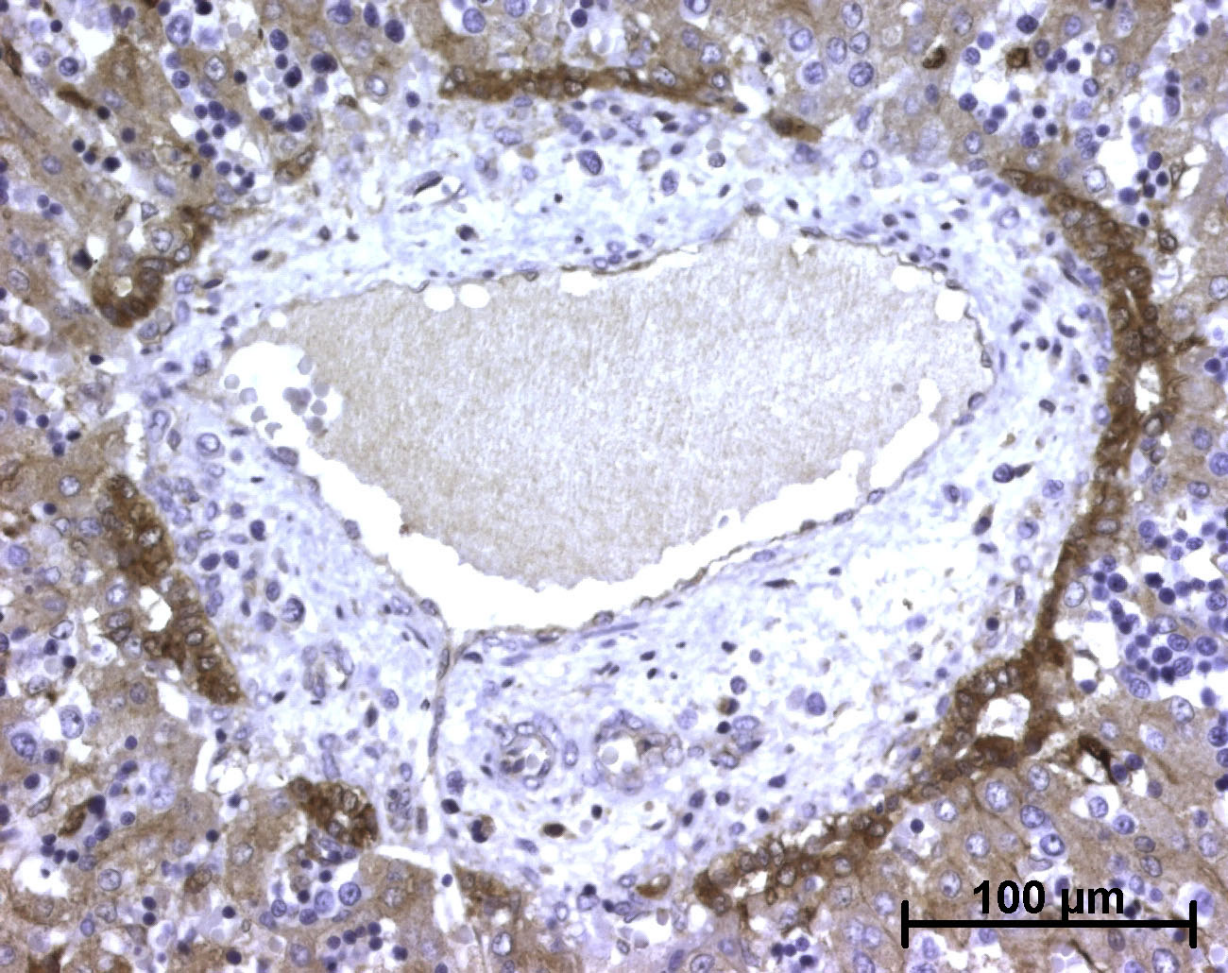
**Cellular retinol-binding protein-1 (CRBP-1) expression in normal fetal liver**. At the beginning of the remodelling stage, biliary structures express CRBP-1 stronger than hepatocytes. The portal stromal cells are not stained (13 WD).

**Figure 13:**
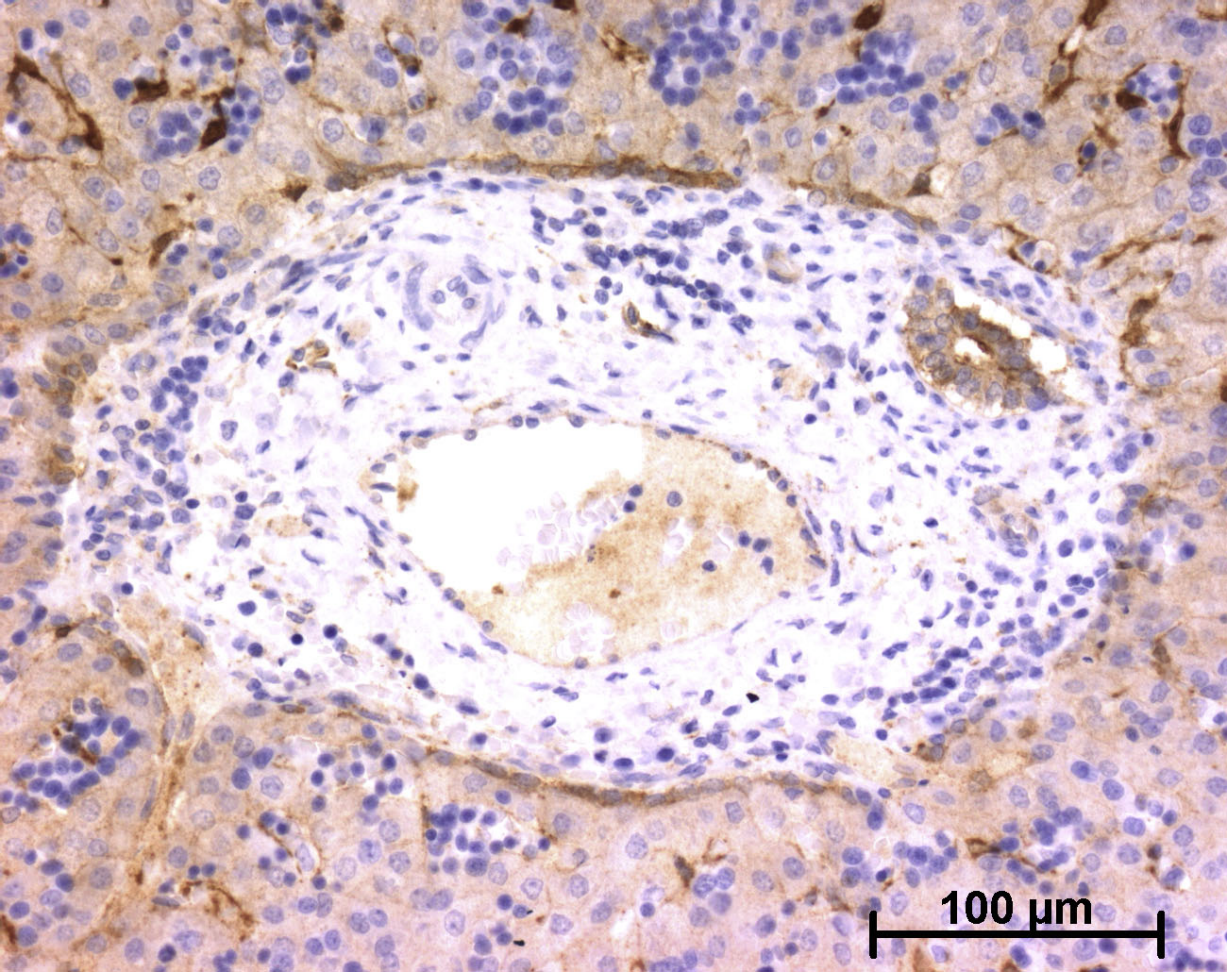
**Cellular retinol-binding protein-1 (CRBP-1) expression in normal fetal liver**. At a late stage of the remodelling stage, biliary structures express CRBP-1 stronger than hepatocytes. The portal stromal cells are not stained (20 WD).

**Figure 14:**
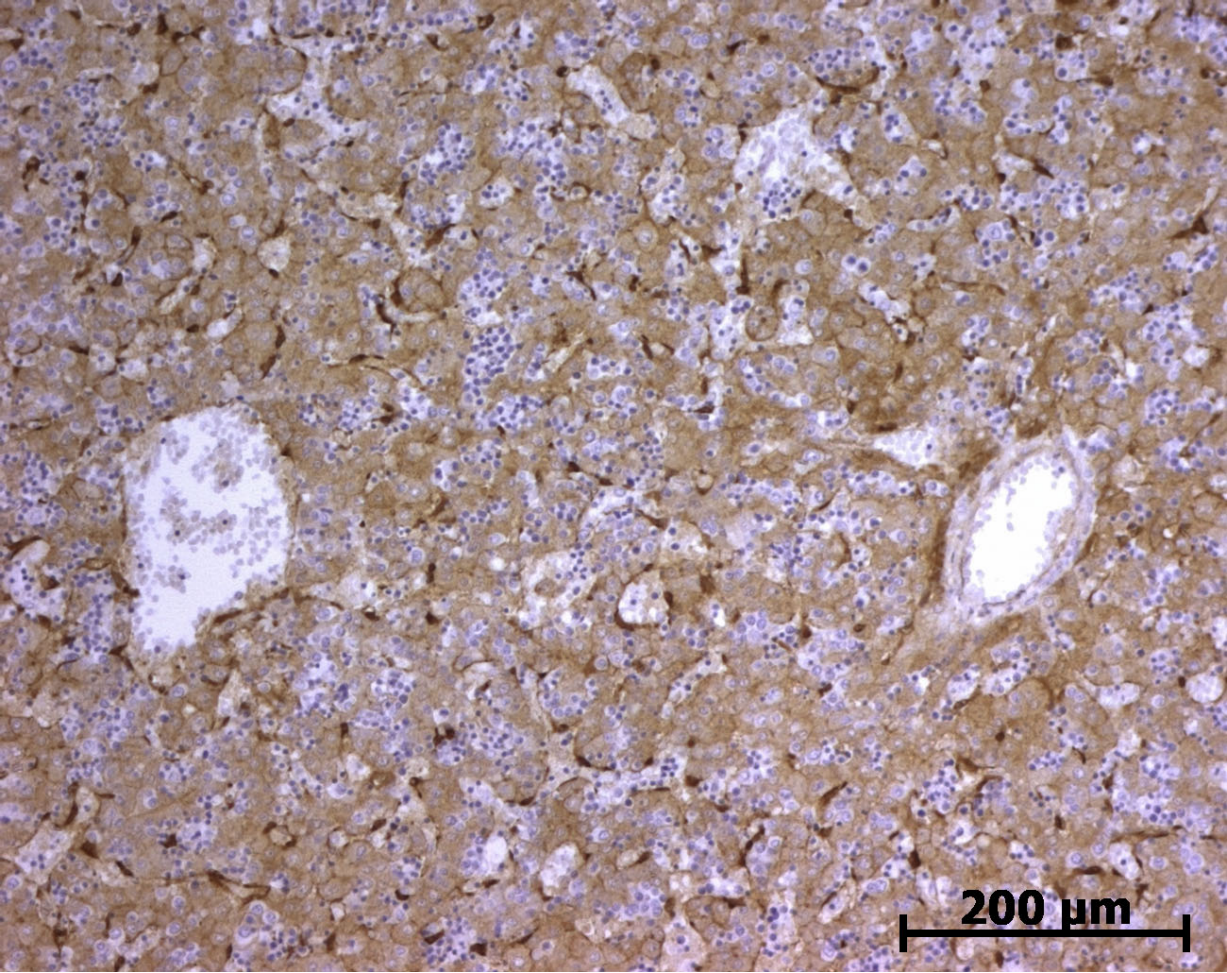
**Cellular retinol-binding protein-1 (CRBP-1) expression in normal fetal liver**. Numerous HSC express CRBP-1 in the parenchyma (11 WD).

**Figure 15:**
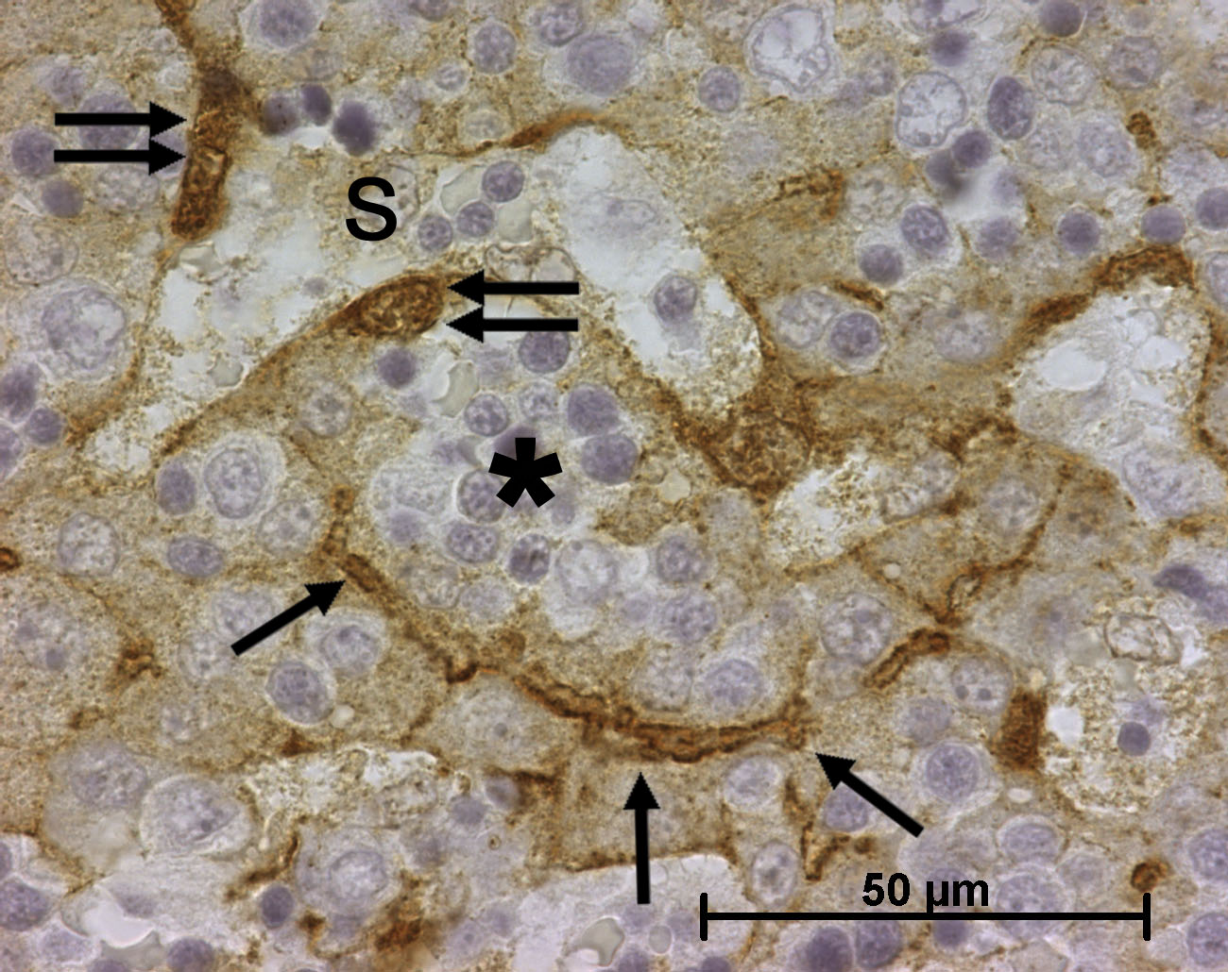
**Cellular retinol-binding protein-1 (CRBP-1) expression in normal fetal liver**. Around the sinusoid (S), CRBP-1 stained HSC (double arrow) are present in the Disse space (*), where haematopoiesis is observed. Hepatocytes express also CRBP-1 with reinforcement in the canaliculi (arrow) (11 WD).

**Figure 16:**
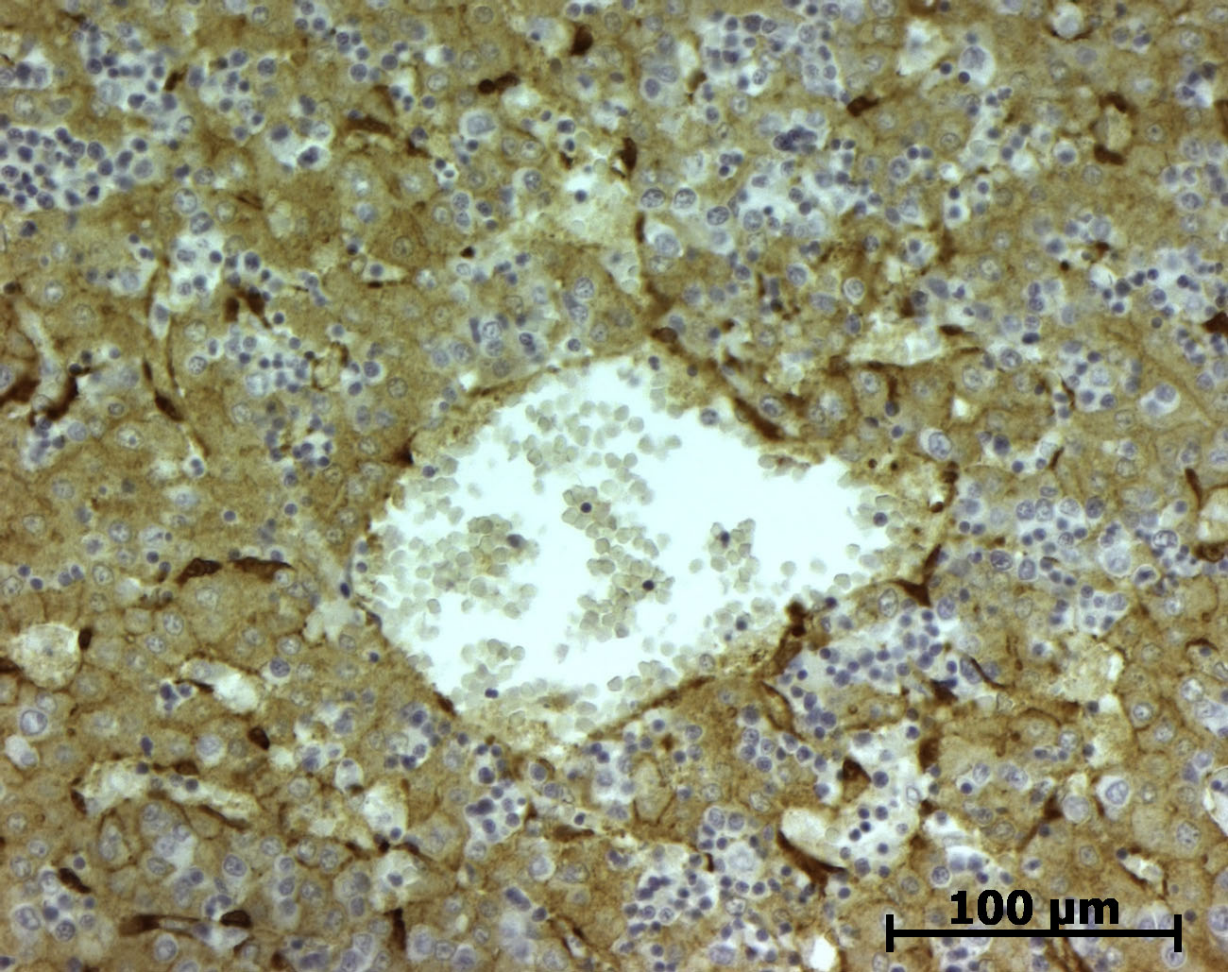
**Cellular retinol-binding protein-1 (CRBP-1) expression in normal fetal liver**. Second layer cells around the centrolobular vein express CRBP-1 (11 WD).

During portal tract development, portal mesenchymal cells never expressed CRBP-1; in contrast biliary cells regularly showed a granular cytoplasmic expression (Figures 12 and 13). This cytoplasmic staining in biliary cells was stronger than in fetal hepatocytes but lower than in the stained cells of the Disse space. In lobular area, until the 13^th ^WD, various number of CRBP-1 stained cells present in the Disse space was observed: no cells in 2 cases, rare cells in 7 cases and numerous cells in 4 cases (Figure 14). After the 13^th ^WD, numerous stained cells were present in all cases, excepted 2 cases where a few cells were observed. Between the 16^th ^WD and the 18^th ^WD, numerous cytoplasmic processes were visible in these CRBP-1 stained cells present in the Disse space. Except in the oldest case, the density of stained cells was lower than in the adult liver. All cases showed a low cytoplasmic CRBP-1 staining in the hepatocytes and canaliculi were often underlined by a reinforcement of the CRBP-1 staining (Figure 15). Fusiform cells around centrolobular veins expressed CRBP-1 (Figure 16).

#### CD34

**Figure 17:**
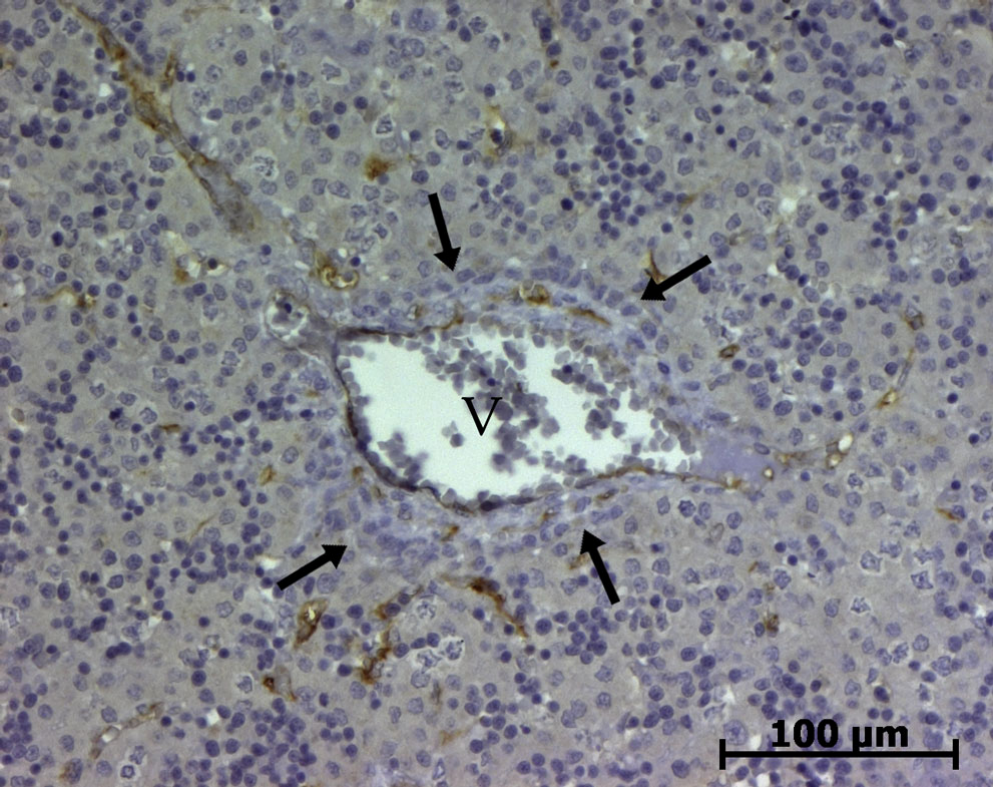
**CD34 expression in normal fetal liver**. At the ductal plate stage, only endothelial of the portal vein (V) or terminal venules express CD34; portal mesenchymal cells as well as ductal plate (arrows) are negative (11 WD).

**Figure 18:**
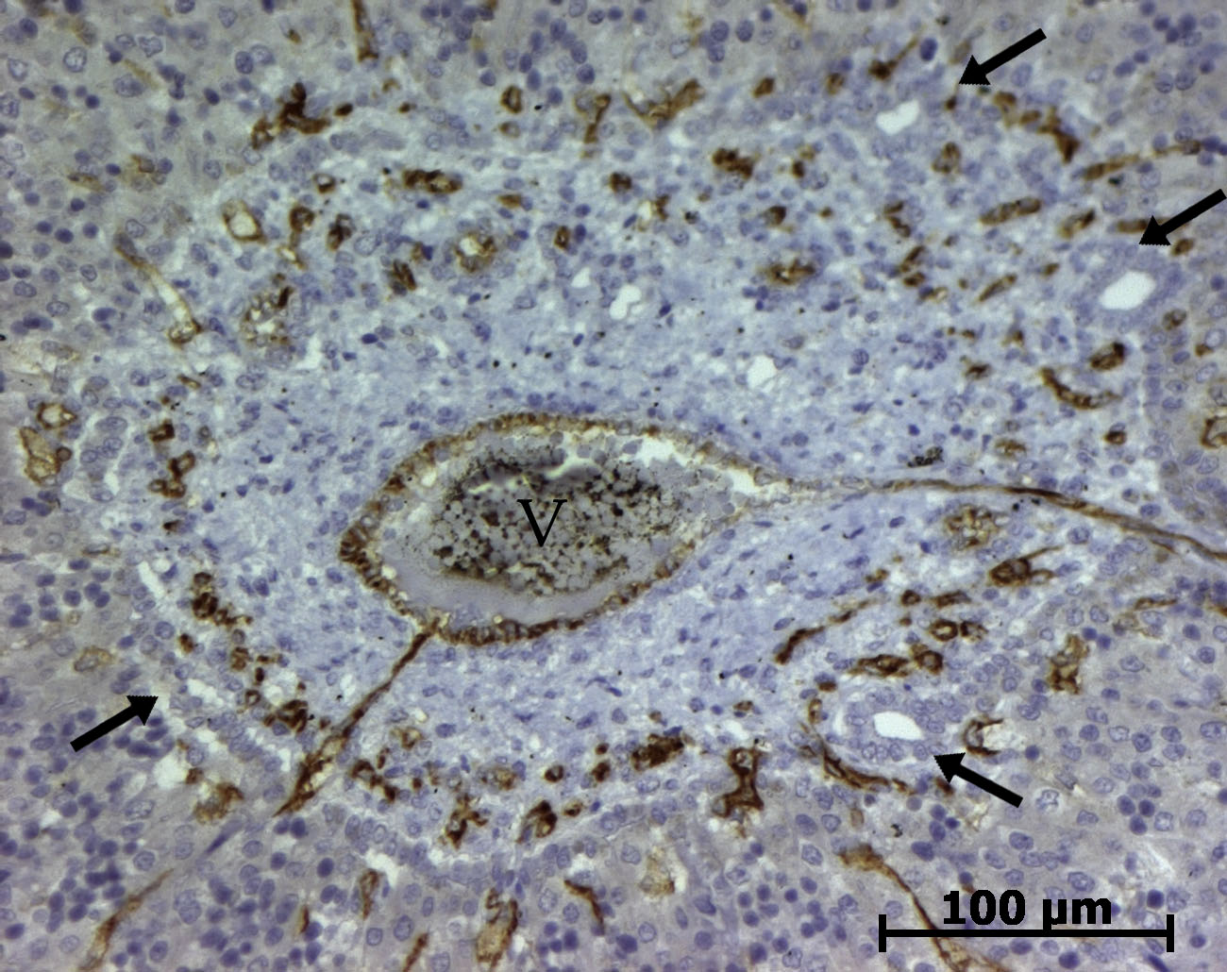
**CD34 expression in normal fetal liver**. At the remodelling stage, endothelial of the portal vein (V), arteries or terminal venules express CD34; portal mesenchymal cells as well as biliary structures (arrows) are negative (11 WD).

**Figure 19:**
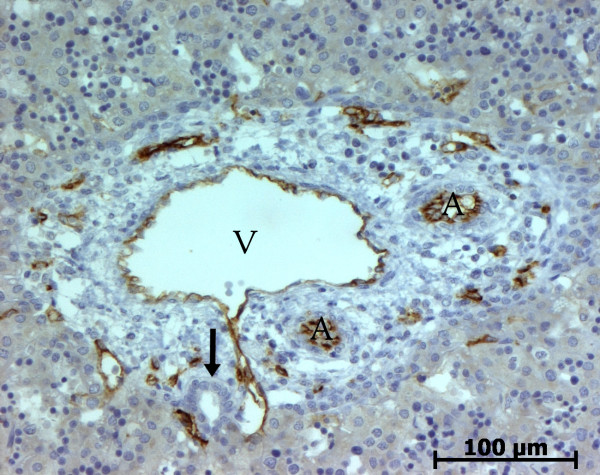
**CD34 expression in normal fetal liver**. At the remodelled stage, endothelial of the portal vein (V), arteries (A) or terminal venules express CD34; portal mesenchymal cells as well as bile duct (arrow) are negative (13 WD).

**Figure 20:**
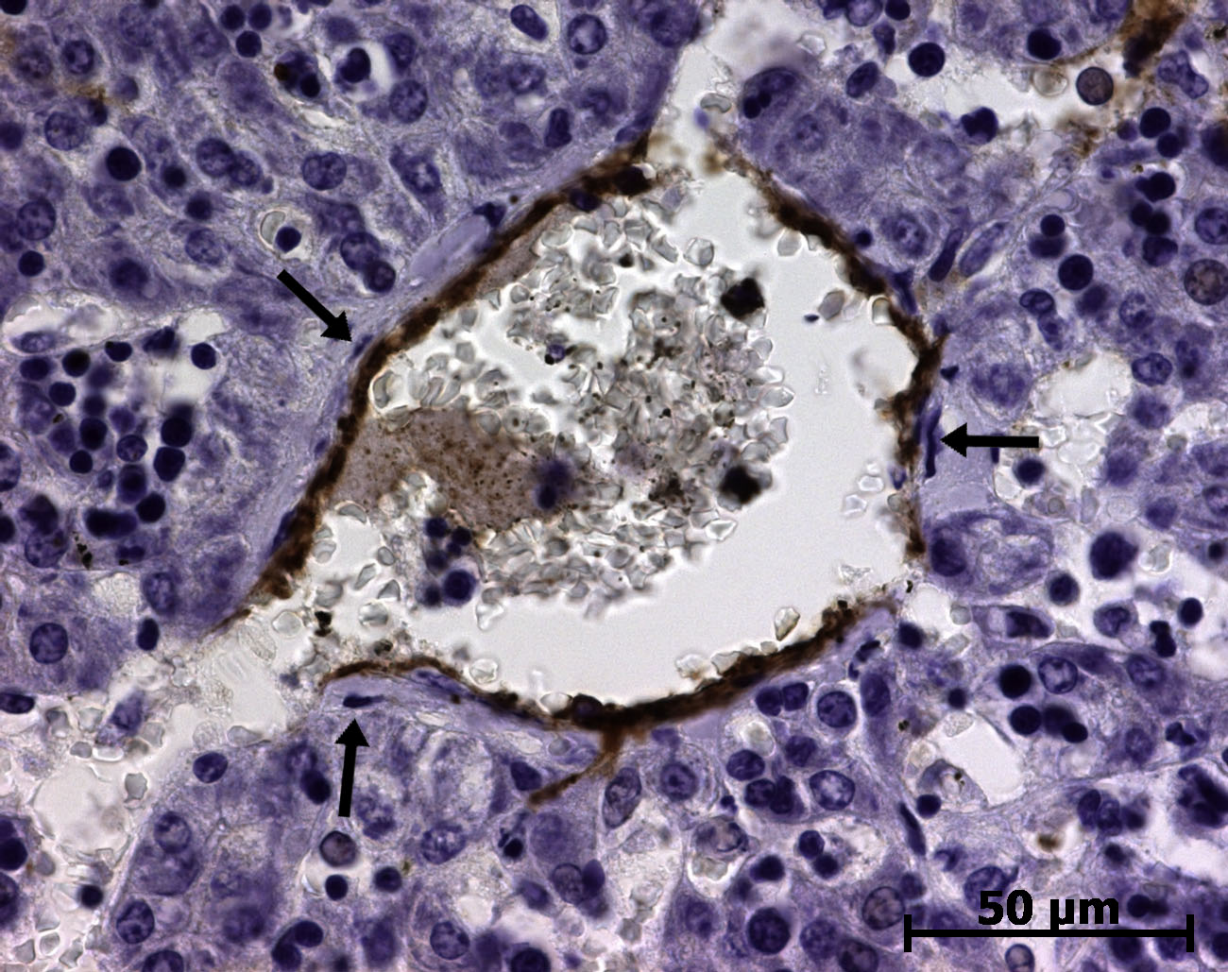
**CD34 expression in normal fetal liver**. Around the centrolobular vein, endothelial cells express CD34. The second layer cells are negative (arrows) (23 WD).

During the maturation of the portal tract, endothelial cells of portal vessels, notably the terminal venules, and centrolobular vein are stained (Figures 17, 18, 19 and 20). No portal mesenchymal cell, hepatocytic cell and sinusoidal cell were stained.

#### Cytokeratin 19

**Figure 21:**
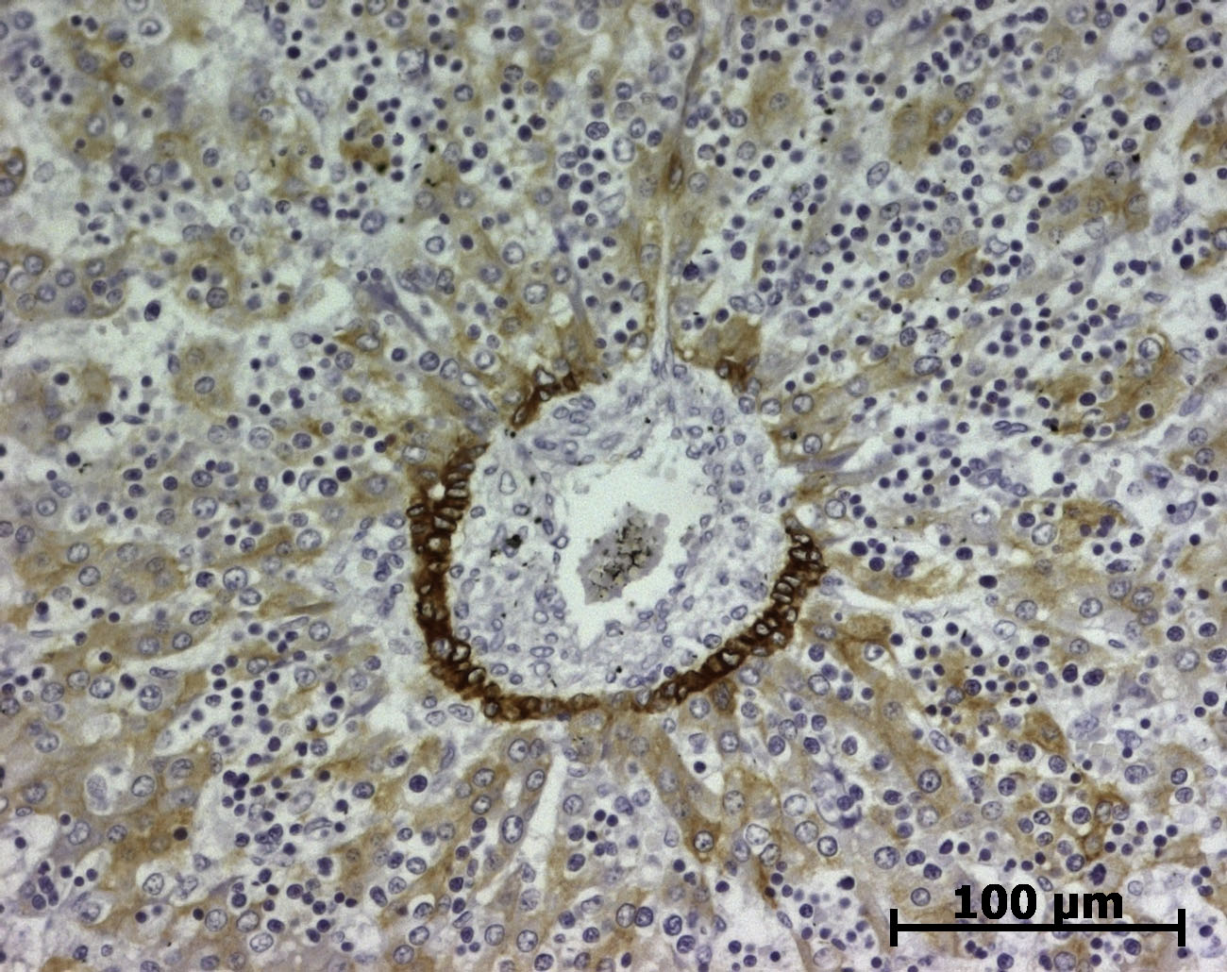
**Cytokeratin 19 expression in normal fetal liver**. At the ductal plate stage, ductal plate express cytokeratine 19 (11 WD).

**Figure 22:**
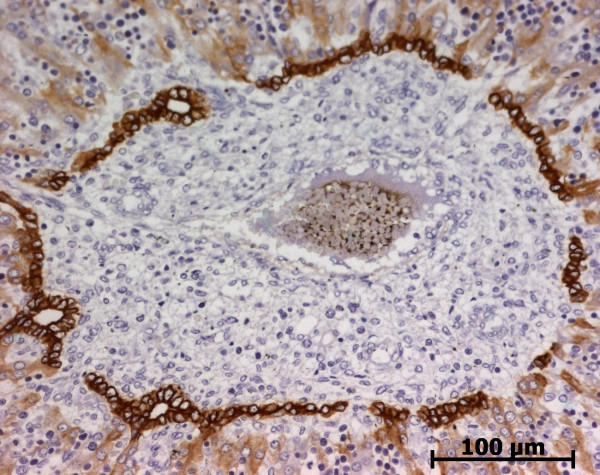
**Cytokeratin 19 expression in normal fetal liver**. At the remodelling stage, biliary structures express cytokeratine 19 (11 WD).

**Figure 23:**
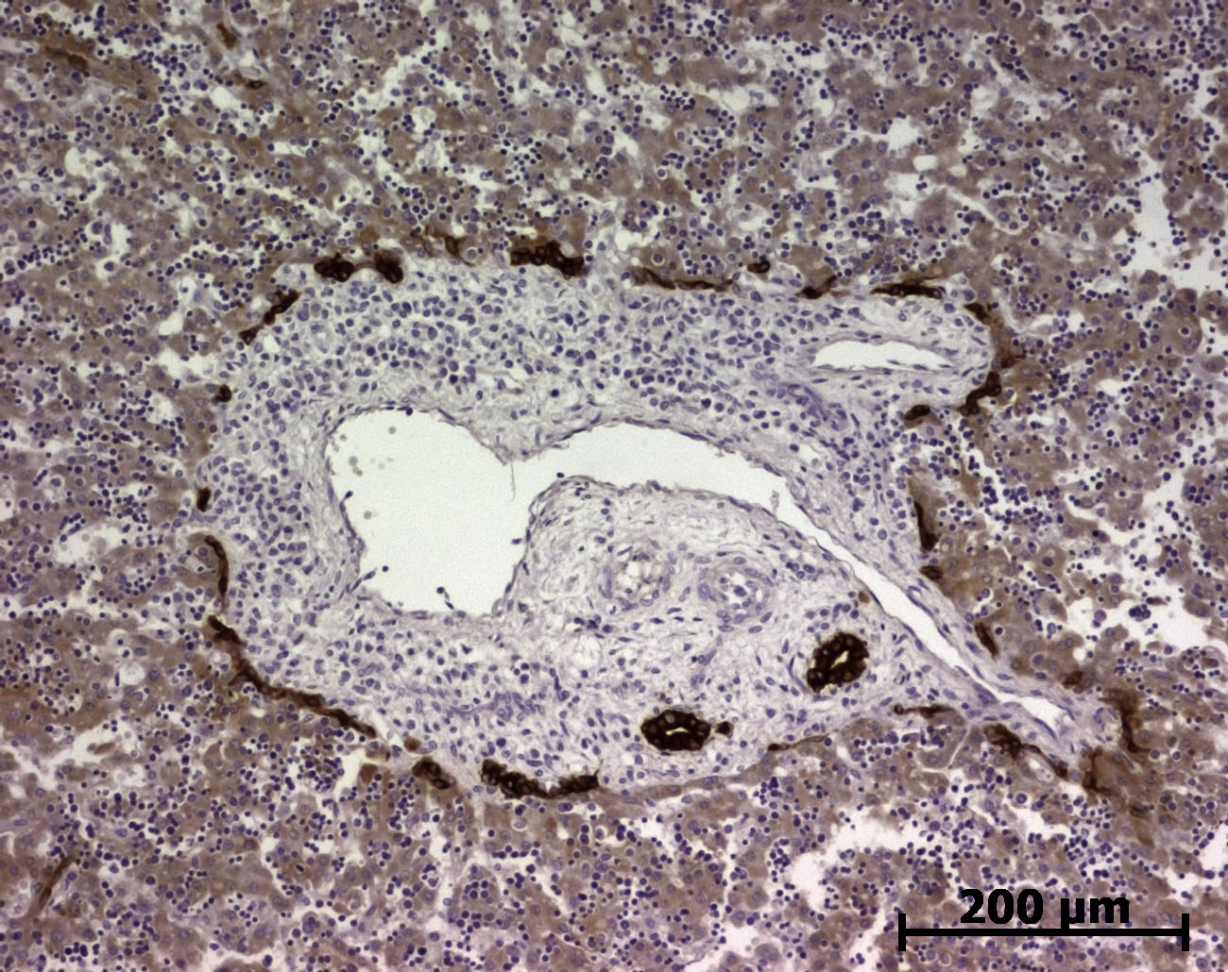
**Cytokeratin 19 expression in normal fetal liver**. At the remodelled stage, biliary structures express cytokeratine 19 (11 WD).

The staining of the biliary cells depended of the level of maturation. At the ductal plate stage, the cells of the ductal plate began to express cytokeratin 19 (Figure 21). During the remodelling of the ductal plate (Figure 22) and at the remodelled stage (Figure 23), the biliary ducts were regularly stained. As previously described [20], there was a weak staining of hepatocytes, principally in the youngest cases. In all cases, all fibrocompetent cells were not stained.

### Fibrous fetal liver – Histology

**Figure 24:**
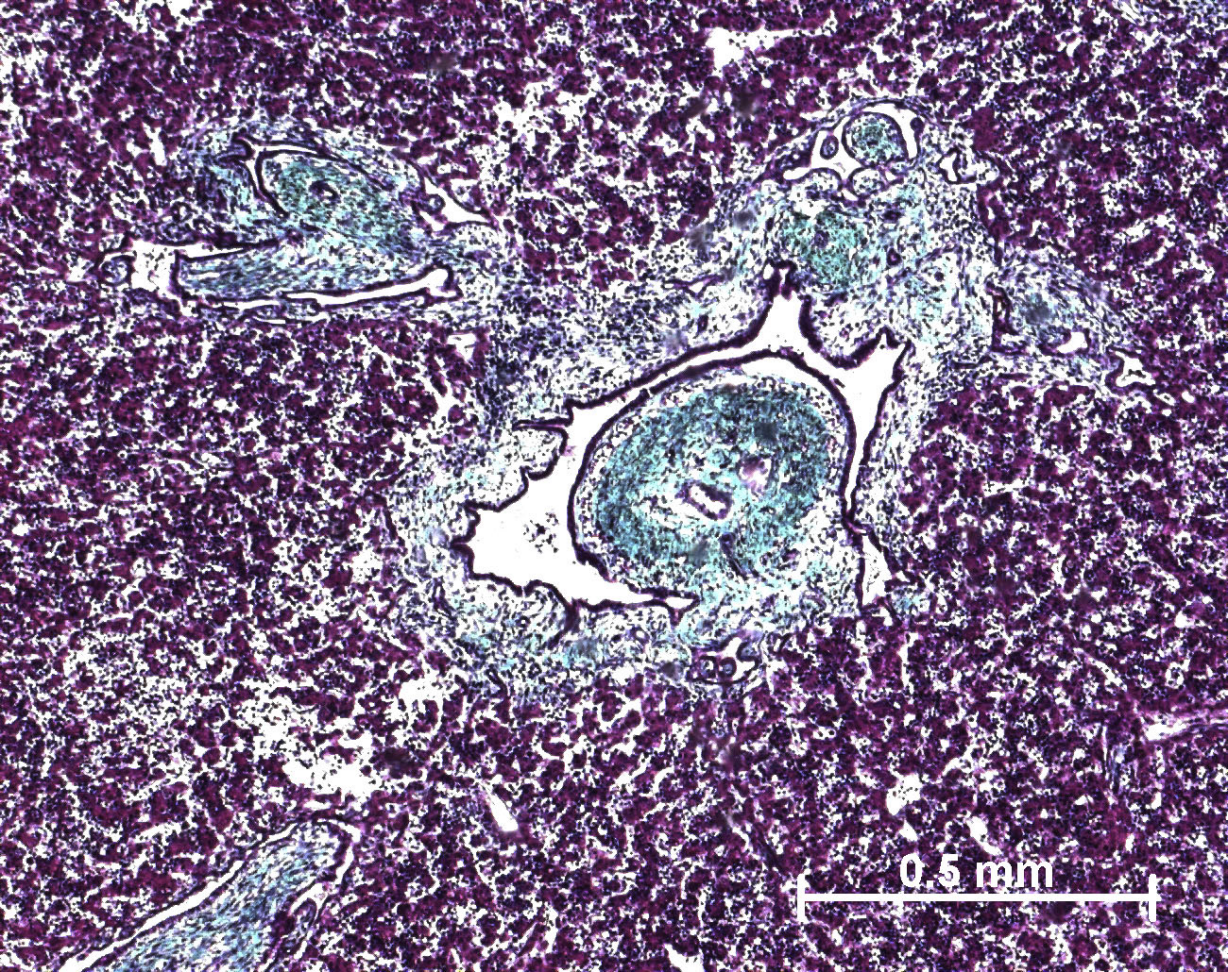
**A case of autosomal recessive polycystic kidney disease**. At a late stage of maturation, portal tract is enlarged by fibrosis and contained numerous abnormal bile ducts (trichrome staining)) (22 WD).

At the beginning of the portal tract development, i.e. ductal plate stage, there were no difference in the portal tract morphology in all pathological livers and normal fetal livers. At the end of the portal tract development, portal tracts were enlarged by fibrosis (Figure 24) with sometimes septa between portal tracts. The circumferential proliferation of bile ducts was low in IDS2, moderate in MKS, and important with dilated bile ducts in ARPKD. In all cases, portal tracts showed a proliferation of fusiform cells around the bile ducts and an increase in the number of hepatic artery branches. The architecture of lobular parenchyma was unchanged.

### Fibrous fetal liver – Immunohistochemistry

#### Alpha-smooth muscle actin (ASMA)

**Figure 25:**
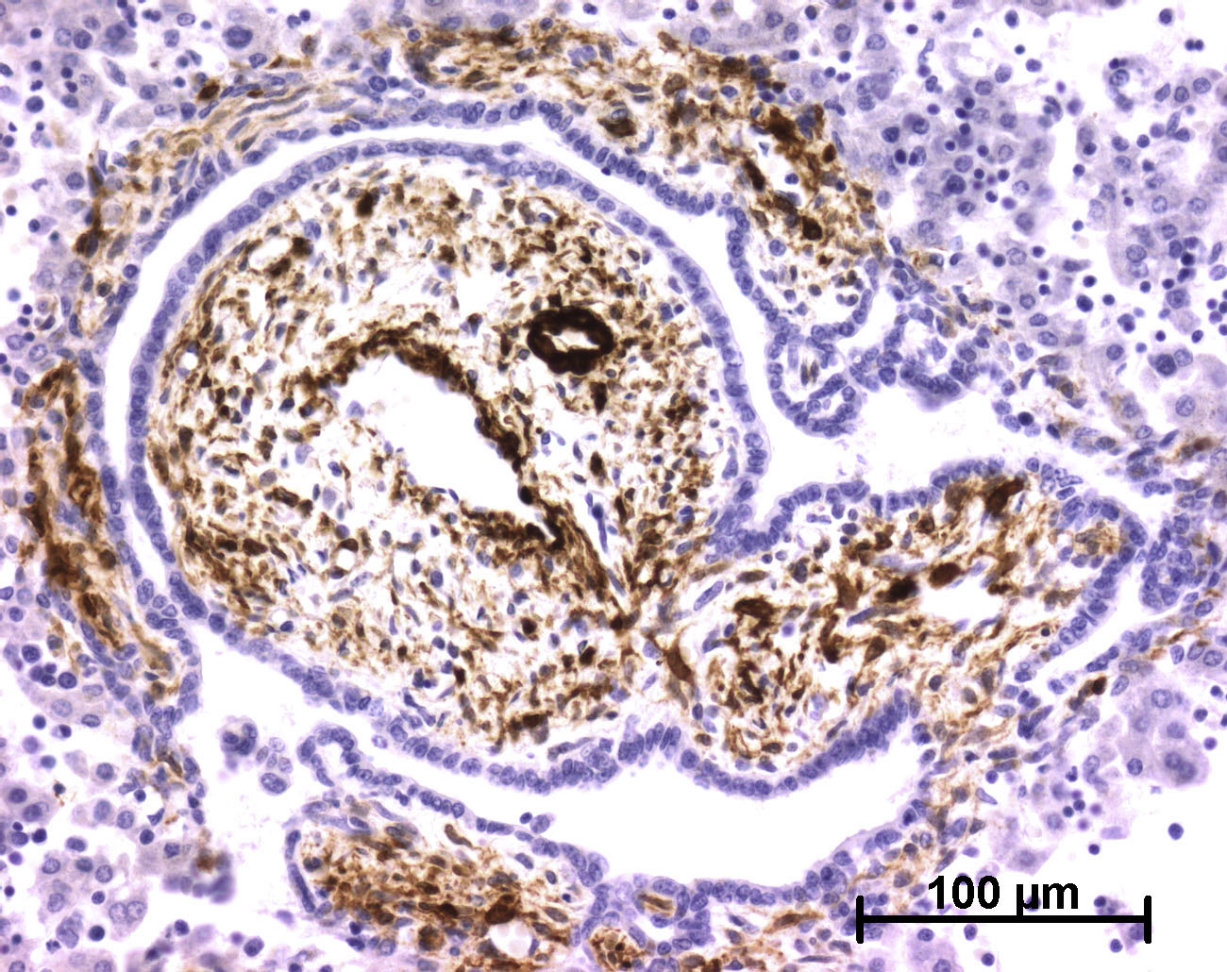
**Alpha-smooth muscle actin (ASMA) expression in a case of autosomal recessive polycystic kidney disease**. As expected, vessels wall cells express ASMA. Abnormal bile ducts are surrounded by ASMA positive stromal cells (22 WD).

In the portal tract, the pattern of ASMA expression was the same as in normal fetal liver at the beginning of portal tract development. At the end of development, when portal tracts were enlarged by fibrosis, numerous fusiform cells surrounding the abnormal bile ducts were stained as well as cells in vascular tunica media (Figure 25). In the lobular area, except in one case of MKS, cells in the Disse space did not express ASMA. Fusiform cells around centrolobular vein expressed ASMA.

#### h-Caldesmon

**Figure 26:**
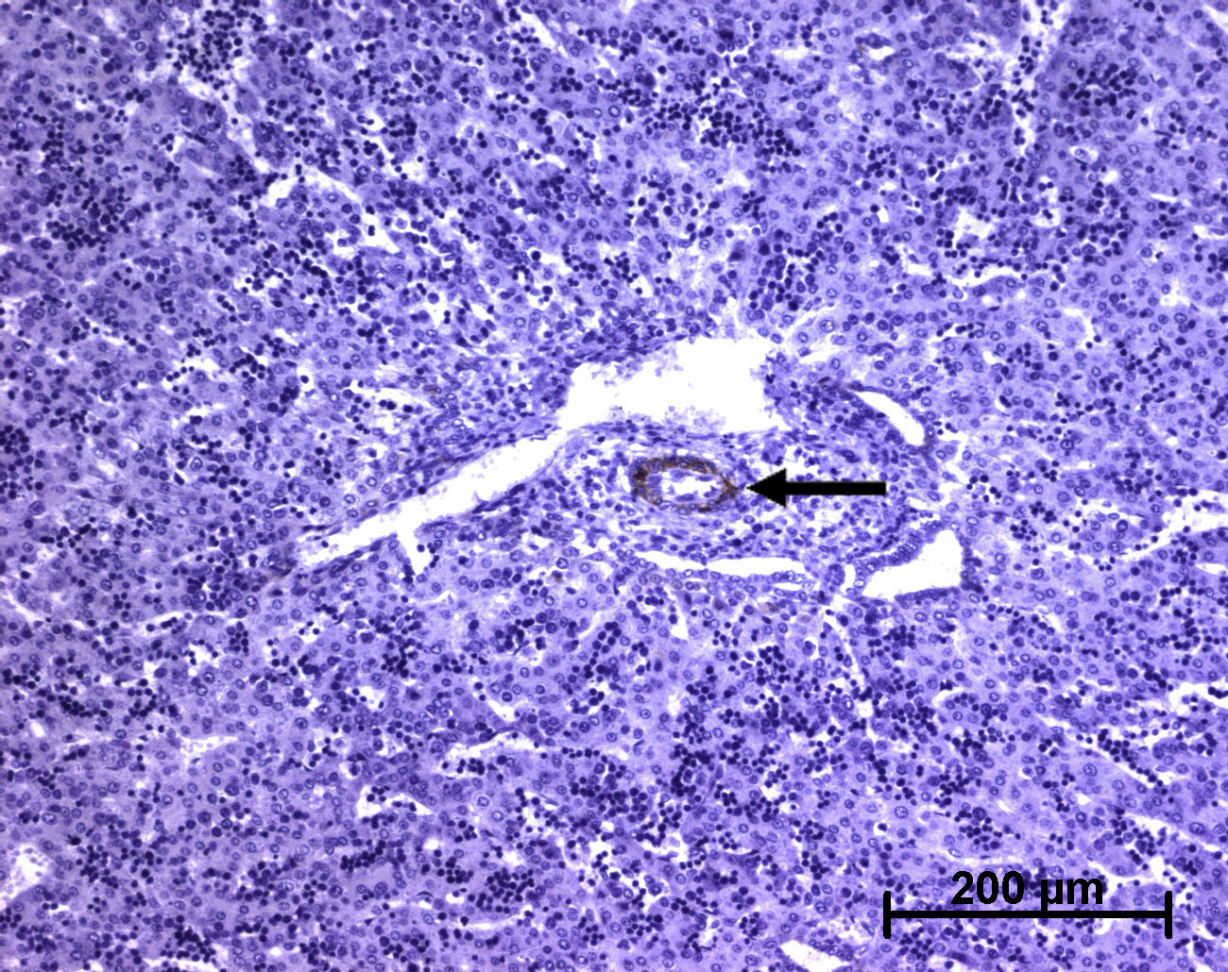
**h-Caldesmon expression in a case of autosomal recessive polycystic kidney disease**. Only arterial tunica media cells (arrow) express h-caldesmon.; ASMA positive cells around abnormal bile ducts do not expressed h-caldesmon (22 WD).

The evolution of h-caldesmon expression pattern was the same as in the normal fetal liver: in all cases, only cells of the arterial tunica media were stained (Figure 26).

#### Cellular retinol-binding protein-1 (CRBP-1)

**Figure 27:**
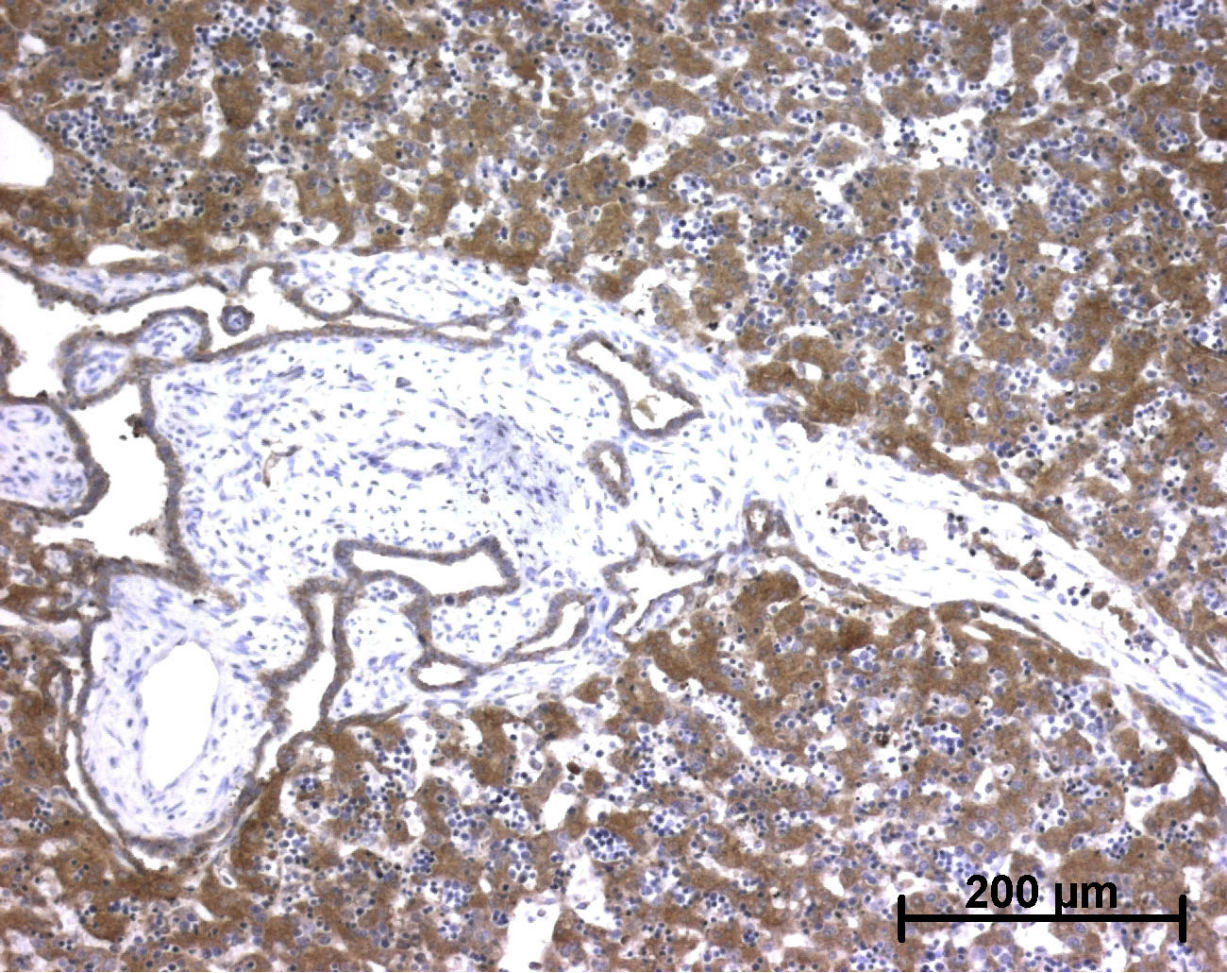
**CRBP-1 expression in a case of autosomal recessive polycystic kidney disease**. Portal stromal cells do not express CRBP-1 (22 WD).

In all cases, portal mesenchymal cells did not express CRBP-1 (Figure 27). In lobular parenchyma, excepted for 3 cases, numerous HSC were stained and exhibited the same pattern of CRBP-1 expression than HSC in the normal fetal liver. CRBP-1 expression pattern of hepatocytes and of biliary cells was the same than in the normal fetal liver.

#### CD34

**Figure 28:**
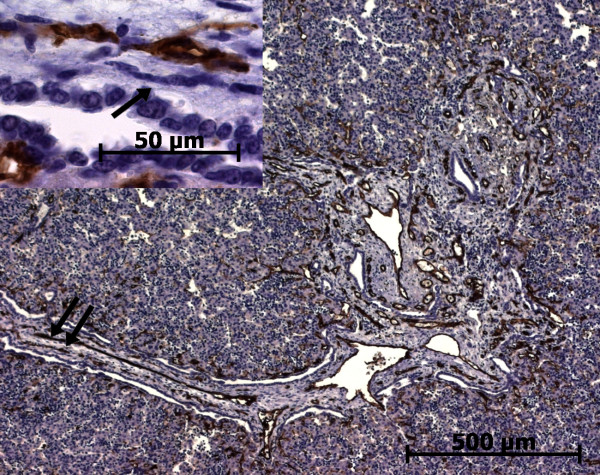
**CD34 expression in a case of autosomal recessive polycystic kidney disease**. Endothelial cells of the vessels enmeshed in the enlarged portal tract, in the fibrous septa or around the biliary structures express CD34; the portal stromal cells do not expressed CD34 (arrow, left insert) (22 WD).

As previously described [12], there are more stained capillaries in the enlarged portal tracts than the normal liver. These stained capillaries are numerous in the fibrous septa and around the biliary structures (Figure 28). The fusiform mesenchymal cells in the portal tract are not stained (Figure 28).

#### Cytokeratin 19

**Figure 29:**
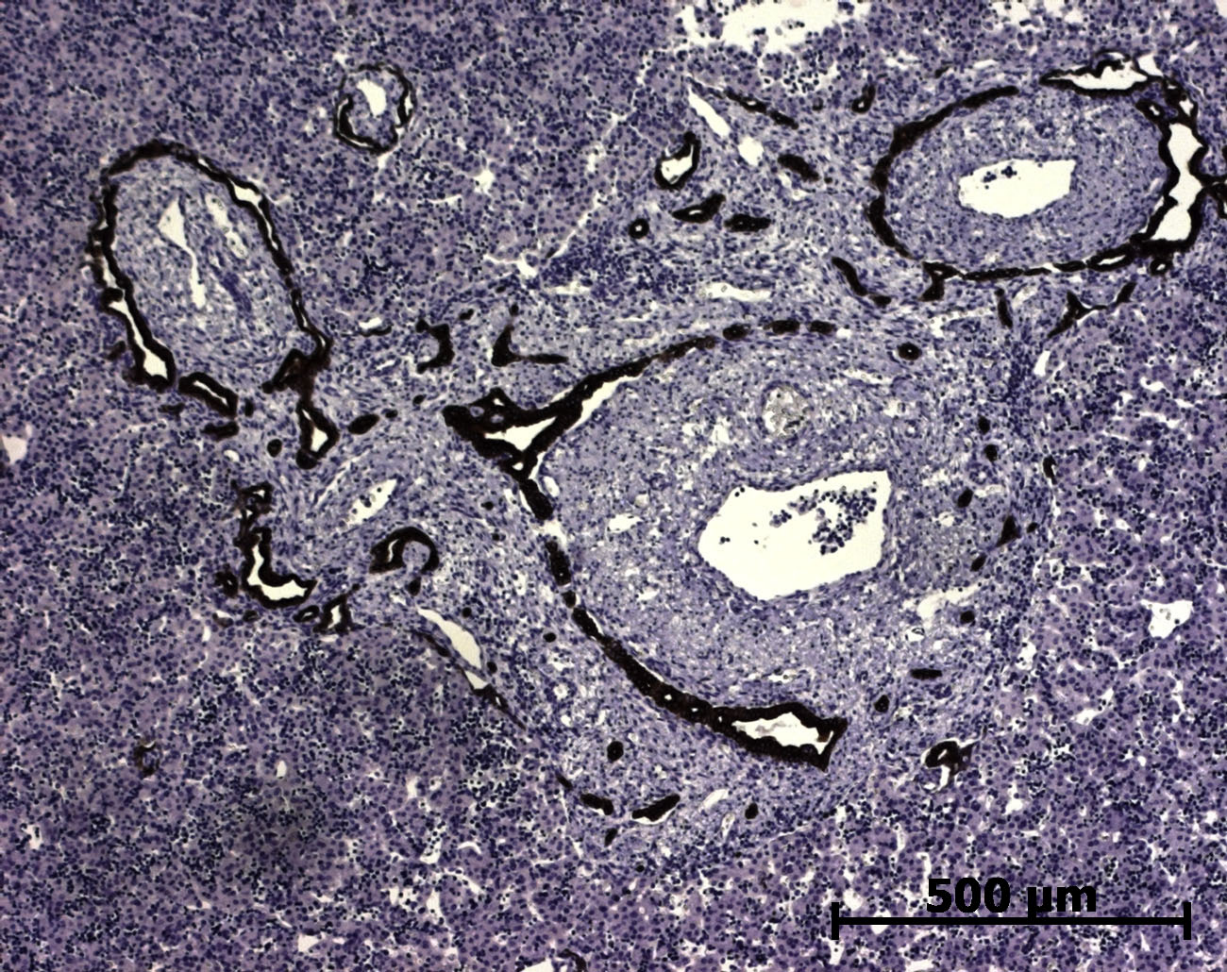
**Cytokeratin 19 expression in a case of autosomal recessive polycystic kidney disease**. Only biliary structures express cytokeratin 19 (22 WD).

The staining of the biliary cells depended of the level of maturation. In the beginning, the cells of the ductal plates began to express cytokeratin 19. During the abnormal remodeling of the ductal plate, the biliary proliferation was regularly stained (Figure 29). In all cases, cells in the Disse space were not stained.

## Discussion

Our study explored the phenotypic heterogeneity of the mesenchymal cells during liver development, mainly along the portal tract tree in normal and in a large series of fibrous fetal liver. For the first time, 3 markers, which are expressed in hepatic stromal cells were used: ASMA, a cytodifferentiated-related contractile protein expressed notably by smooth muscle cells and myofibroblasts, and 2 others markers poorly used in fetal liver studies, h-caldesmon (150 kDa caldesmon), an isotype of caldesmon expressed by smooth muscle cells, and CRBP-1 which is involved in vitamin A metabolism and is highly expressed in HSC [3,6,9,19].

In the normal fetal liver, phenotypic changes of the portal mesenchymal cells are observed during the 3 stages of the portal tract maturation. At the ductal plate stage, all the mesenchymal cells expressed ASMA and did not expressed CRBP-1 or h-caldesmon. At the remodelling stage, a fibroblastic subpopulation of cells were negative for the 3 markers cited above, but were positive for vimentin, appeared in the middle area of the portal tract at distance from vessels and biliary structures. At the remodelled stage, only cells of arterial tunica media expressed ASMA and h-caldesmon and displayed a smooth muscle phenotype. The cells of portal vein tunica media expressed ASMA, but not h-caldesmon. As reported in adult liver, the connective tissue of the portal tract contained fibroblastic cells, also called portal fibroblasts, which expressed vimentin but not ASMA, CRBP-1 or h-caldesmon [3,4]. During the maturation of the portal tract in normal fetal liver, ASMA expressing mesenchymal cells around future portal vein, called myofibroblasts by Libbrecht et al. [12], were replaced or could result from the differentiation into portal fibroblasts and contractile cells of the portal vein tunica media. The sequential involvement of myofibroblastic cells during fetal development was also observed in other organs, notably in cardiac valve or lung [21,22]. Concerning the portal vein, we hypothesize that contractile cells in the tunica media could achieve their differentiation after the birth into smooth muscle cells because, in adult normal liver, some cells present in the thin tunica media of portal vein expressed h-caldesmon (data not shown), a more specific and late marker of smooth muscle cell differentiation [6]. We can speculate that this maturation of portal vein smooth muscle cells is related to the change of the portal venous circulation in the liver at birth. Unlike portal vein, the tunica media cells of the hepatic artery branches which were appeared during the remodelling stage, were early completely differentiated into smooth muscle cells, expressing regularly ASMA as well as h-caldesmon. These smooth muscle cells of the tunica media might take origin from the tunica media cells of the upstream arteries. However, we cannot exclude that they differentiate from the portal myofibroblasts.

IDS2, MKS and ARPKD are autosomal recessively inherited disorders characterised in the liver by abnormal development of the portal tract and notably ductal plate malformation [14-16]. In these diseases, the portal tract stroma is enlarged by fibrosis and contained more stromal cells. As described previously in one case of MKS [17], we showed that, in all our pathological cases, a myofibroblastic subpopulation, which expressed only ASMA persists during all the abnormal maturation of the portal tract and is condensed around the abnormal biliary structures. These myofibroblasts which were present in all portal tracts whatever the calibre of bile ducts and not only in the larger-calibre septal bile ducts, as seen in the normal liver until 2 years of age [12], were probably responsible of the excessive deposition of portal extracellular matrix. This myofibroblastic reaction resembles that seen in human liver diseases affecting bile ducts or in experimental models such as bile duct ligation. However, in these cases, myofibroblasts surrounding the ductular proliferation seemed to derive from the transdifferentiation of portal fibroblasts [23-26].

In the lobular area, the development was the same in all our normal and pathological cases. We showed that HSC are present early in the Disse space and express CRBP-1. The CRBP-1 staining showed that the thin cytoplasmic processes are poorly developed in the beginning and become more important later. CRBP-1 expressing HSC play a pivotal role in intrahepatic uptake, storage and release of retinoids [27]. As previously described, our study in fetal liver showed that the number of CRBP-1 expressing HSC was variable but gradually increased with the age of development [9,28]. As shown here, CRBP-1 was also expressed all along the biliary tree from canaliculi to extrahepatic bile duct; and this expression was reinforced on the apical/luminale membrane. The bile acid synthesis begins at about 5–9 WD and its secretion at about 12 WD. Bile contains retinoids [29]. We assume that, besides the blood retinol transport, there is a biliary transport of retinoids [3].

## Conclusion

Our study shows that, during the portal tract development, the portal mesenchymal cells are involved in a morphological phenotypic shift from myofibroblasts to portal fibroblasts and vascular smooth muscle cells; in case of portal fibrosis following ductal plate malformation, portal myofibroblasts persist around the abnormal biliary structures.

## Methods

### Human fetal liver specimens

Normal (28 cases, table 1) and pathological (11 cases, table 2) human fetal tissues were obtained from spontaneous or therapeutic/medical abortion performed in compliance with the French legislation. The causes of fetal death, sex, abnormalities after the autopsy and age according to the date of last menstrual period were summarized in tables 1 and 2. Developmental stages, indicated in weeks after conception, were estimated from the menstrual history and confirmed on anatomic criteria using a regression equation for predicting fetal age [30]. The procedures were in accordance with the European Guidelines for the use of human tissues.

**Table 1:** Clinical data of non-pathological livers – Part I.

	Estimation of gestional age/Date of last menstrual period	Sex	Cause of fetus death	Pathology
1	-/9 WA	-	Medical abortion	Extra-uterine pregnancy
2	11 WD/13 WA	M	Spontaneous abortion	Infection
3	11 WD/13 WA	F	Medical abortion	Trisomy 18
4	11 WD/13 WA	M	Medical abortion	Amniotic bridle
5	11 WD/13 WA	M	Spontaneous abortion	Infection
6	11 WD/13 WA	F	Medical abortion	Trisomy 21
7	11 WD/13 WA	M	Medical abortion	Cervical hygroma
8	11 WD/13 WA	M	Medical abortion	Encephalocele
9	12 WD/14 WA	M	Medical abortion	Uro-genital abnormality
10*	13 WD/15 WA	F	Spontaneous abortion	-
11*	13 WD/15 WA	M	Spontaneous abortion	-
12	13 WD/15 WA	M	Spontaneous abortion	Muscular dystrophy
13	13 WD/15 WA	F	Spontaneous abortion	Trisomy 18
14	16 WD/18 WA	M	Spontaneous abortion	-
15	16 WD/18 WA	F	Medical abortion	Trisomy 21
16	17 WD/19 WA	M	Medical abortion	Trisomy 21
17	18 WD/20 WA	M	Spontaneous abortion	Infection
18	18 WD/20 WA	F	Medical abortion	Visceral abnormalities
19	20 WD/22 WA	M	Medical abortion	Retroplacental hematoma
20	20 WD/22 WA	F	Medical abortion	Visceral abnormalities
21	20 WD/22 WA	M	Medical abortion	Premature membranes rupture
22	21 WD/23 WA	M	Medical abortion	Visceral abnormalities
23	21 WD/23 WA	F	Medical abortion	Visceral abnormalities
24	23 WD/25 WA	F	Spontaneous abortion	Infection
25	23 WD/25 WA	F	Medical abortion	-
26	23 WD/25 WA	F	Medical abortion	Nanism
27	27 WD/29 WA	M	Spontaneous abortion	Rupture of the uterine corpus
28	31 WD/33 WA	M	Stillborn foetus	Anasarca

*: Cases 10 and 11 were twins. WD: weeks of development; WA: weeks of amenorrhea; M: male; F: female.

**Table 2:** Clinical data of pathological livers – Part II.

	Estimation of gestional age/Date of last menstrual period	Sex	Cause of fetus death	Pathology
29	15 WD/17 WA	F	Medical abortion	IDS2
30	19 WD/21 WA	M	Medical abortion	IDS2
31	36 WD/38 WA	F	Neonatal death	IDS2
32	13 WD/15 WA	M	Medical abortion	MKS
33	13 WD/15 WA	F	Medical abortion	MKS
34	15 WD/17 WA	F	Medical abortion	MKS
35	16 WD/18 WA	M	Medical abortion	MKS
36	22 WD/24 WA	M	Medical abortion	MKS
37	13 WD/15 WA	M	Medical abortion	ARPKD
38	22 WD/24 WA	M	Medical abortion	ARPKD
39	22 WD/24 WA	F	Medical abortion	ARPKD

WD: weeks of development; WA: weeks of amenorrhea; M: male; F: female. IDS2: Ivemark's dysplasia syndrome type 2 – MKS: Meckel-Gruber syndrome – ARPKD: autosomal recessive polycystic kidney disease

The tissue samples were routinely formalin fixed and paraffin embedded; five μm-thick paraffin sections were performed and stained with haematoxylin-eosin-saffron (HES) for diagnosis purposes. Additional sections were stained with Masson's trichrome or used for immunohistochemistry.

### Immunohistochemistry

The immunohistochemical study was routinely performed using an automated immunostainer (Dako A/S, Glostrup, Denmark) with mouse monoclonal primary antibodies against ASMA (1/100, Dako), CRBP-1 (1/100 [31]), h-caldesmon (1/50, Dako), CD34 (Dako), cytokeratine 7 (Dako), and cytokeratin 19 (Dako). The epitopes were detected with the Envision+ system horseradish peroxidase detection kit and revealed with liquid diaminobenzidine (Dako).

For double immunofluorescence, slides were incubated with mouse antibody against vimentin (1/800, Dako) and rabbit antibody against ASMA (1/50, Abcam, Cambridge, UK). Alexa Fluor 568 goat anti-mouse (1/200, Invitrogen, Carlsbad, CA) and Alexa Fluor 488 goat anti-rabbit (1/200, Invitrogen,) were used for the second step.

Sections were examined with a Zeiss Axioplan 2 microscope (Carl Zeiss Microscopy, Jena, Germany) equiped with epiillumination and specific filters. Images were acquired with an AxioCam camera (Carl Zeiss Vision, Hallbergmoos, Germany) by means of the AxioVision image processing and analysis system (Carl Zeiss Vision).

## Competing interests

The authors declare that they have no competing interests.

## Authors' contributions

JV participated in the histological experiments. FPN gave a fetopathology's expertise. CC participated in the histological experiments. DC gave a fetopathology's expertise. CC participated in the design of immunohistochemical study. JR gave his expertise on fibrogenesis. CB and PBS gave a hepatopathology's expertise. SL was responsible for the conception, performed the immunohistochemical study and wrote the manuscript. All authors have read and approved the final manuscript.

## References

[B1] Guyot C, Lepreux S, Combe C, Doudnikoff E, Bioulac-Sage P, Balabaud C, Desmoulière A (2006). Hepatic fibrosis and cirrhosis: The (myo)fibroblastic cell subpopulations involved. Int J Biochem Cell Biol.

[B2] Schmitt-Gräff A, Krüger S, Bochard F, Gabbiani F, Denk H (1991). Modulation of alpha smooth muscle actin and desmin expression in perisinusoidal cells in normal and diseased human liver. Am J Pathol.

[B3] Lepreux S, Bioulac-Sage P, Gabbiani G, Sapin V, Housset C, Rosenbaum J, Balabaud C, Desmoulière A (2004). Cellular retinol-binding protein-1 expression in normal and fibrotic/cirrhotic human liver: different patterns of expression in hepatic stellate cells and (myo)fibroblast subpopulations. J Hepatol.

[B4] Van Rossen E, Borght S Vander, Van Grunsven L, Reynaert H, Bruggeman V, Blomhoff R, Roskams T, Geerts A (2009). Vinculin and cellular retinol-binding protein-1 are markers for quiescent and activated hepatic stellate cells in formalin-fixed paraffin embedded human liver. Histochem Cell Biol.

[B5] Nakayama H, Enzan H, Yamamoto M, Miyazaki E, Yasui W (2004). High molecular weight caldesmon positive stromal cells in the capsule of hepatocellular carcinomas. J Clin Pathol.

[B6] Frid M, Shekhonin B, Koteliansky V, Glukhova M (1992). Phenotypic changes of human smooth muscle cells during development: late expression of heavy caldesmon and calpontin. Dev Biol.

[B7] Nouchi T, Tanaka Y, Tsukada T, Sato C, Marumo F (1991). Appearance of alpha-smooth-muscle-actin-positive cells inhepatic fibrosis. Liver.

[B8] Roskams T, Desmet V (2008). Embryology of extra- and intrahepatic bile ducts, the ductal plate. Anat Rec (Hoboken).

[B9] Geerts A (2001). History, heterogeneity, developmental biology, and functions of quiescent hepatic stellate cells. Semin Liver Dis.

[B10] Cassiman D, Barlow A, Borght S Vander, Libbrecht L, Pachnis V (2006). Hepatic stellate cells do not derive from neural crest. J Hepatol.

[B11] Clotmann F, Libbrecht L, Gresh L, Yaniv M, Roskams T, Rousseau G, Lemaigre F (2003). Hepatic artery malformations associated with a primary defect in intrahepatic bile duct development. J Hepatol.

[B12] Libbrecht L, Cassiman D, Desmet V, Roskams T (2002). The correlation between portal myofibroblasts and development of intrahepatic bile ducts and arterial branches in human liver. Liver.

[B13] Crawford A, Lin X, Crawford J (1998). The normal adult human liver biopsy: a quantitative reference standard. Hepatology.

[B14] Salonen R (1984). The Meckel syndrome: clinicopathological findings in 67 patients. Am J Med Genet.

[B15] Torra R, Alos L, Ramos J, Estivill X (1996). Renal-hepatic-pancreatic dysplasia: an autosomal recessive malformation. J Med Genet.

[B16] Bendon R (1999). Ivemark's renal-hepatic-pancreatic dysplasia: analytic approach to a perinatal autopsy. Pediatr Dev Pathol.

[B17] Kuroda N, Ishiura Y, Kawashima M, Miyazaki E, Hayashi Y, Enzan H (2004). Distribution of myofibroblastic cells in the liver and kidney of Meckel-Gruber syndrome. Pathol Int.

[B18] Wu H, Tao L, Cramer H (1996). Vimentin-positive spider-shaped Kupffer cells. A new clue to cytologic diagnosis of primary and metastatic hepatocellular carcinoma by fine needle aspiration biopsy. Am J Clin Pathol.

[B19] Ceballos K, Nielsen G, Selig M, O'Connell J (2000). Is anti-h-caldesmon useful for distinguishing smooth muscle and myofibroblastic tumors?. Am J Clin Pathol.

[B20] Desmet V, Van Eyken P, Sciot R (1990). Cytokeratins for probing cell lineage relationships in developing liver. Hepatology.

[B21] Shworak N (2004). Angiogenic modulators in valve development and disease: does valvular disease recapitulate developmental signaling pathways?. Curr Opin Cardiol.

[B22] Leslie K, Mitchel J, Woodcock-Mitchell J, Low R (1990). Alpha smooth muscle actin expression in developing and adult human lung. Differentiation.

[B23] Tang L, Tanaka Y, Marumo F, Sato C (1994). Phenotypic change in portal fibroblasts in biliary fibrosis. Liver.

[B24] Tuchweber B, Desmoulière A, Bochaton-Piallat M, Rubbia-Brant L, Gabbiani G (1996). Proliferation and phenotypic modulation of portal fibroblasts in the early stages of cholestatic fibrosis in the rat. Lab Invest.

[B25] Desmoulière A, Darby I, Monte Alto Costa A, Raccurt M, Tuchweber B, Sommer P, Gabbiani F (1997). Extracellular matrix deposition, lysyl oxydase expression, and myofibroblastic differentiation during the initial stages of cholestatic fibrosis in the rat. Lab Invest.

[B26] Lamireau T, Dubuisson L, Lepreux S, Bioulac-Sage P, Fabre M, Rosenbaum J, Desmoulière A (2002). Abnormal hepatic expression of fibrillin-1 in children with cholestasis. Am J Surg Pathol.

[B27] Blomhoff R, Wake K (1991). Perisinusoidal stellate cells of the liver: important roles in retinol metabolism and fibrosis. FASEB J.

[B28] Enzan H, Himeno H, Hiroi M, Kiyoku H, Saibara T, Onishi S (1997). Development of hepatic sinusoidal structure with special reference to the Ito cells. Microsc Res Tech.

[B29] Leo M, Ahmed S, Aleynik S, Siegel J, Kasmin F, Lieber C (1995). Carotenoids and tocopherols in various hepatobiliary conditions. J Hepatol.

[B30] Hadlock F, Deter R, Harrist R, Park S (1984). Estimating fetal age: computer-assisted analysis of multiple fetal growth parameters. Radiology.

[B31] Van Beneden K, Geers C, Van Grunsven L, Pauwels M, Desmoulière A, Verbeelen D, Geerts A, Branden C Van den (2008). CRBP-1 in the renal tubulointerstitial compartment of heathly rats and rats with renal fibrosis. Nephrol Dial Transplant.

